# Comparative Label-Free Proteomics of a High-Glucose Syncytialised Trophoblast Model Identifies Mitochondrial Fission Factor (MFF) as a Candidate Serum Biomarker Associated with Hyperglycaemic Pregnancy

**DOI:** 10.3390/cells15212037

**Published:** 2026-11-09

**Authors:** Amarish Kumar Sharma, Chaitanya Yalavarthi, Pooja Savithri, Gaddipati Giridhar Rahul, Manoj Kumar Jena

**Affiliations:** 1 Department of Biotechnology, School of Bioengineering and Biosciences, Lovely Professional University, Phagwara 144411, Punjab, India; 2 TestGo, The Colony, TX 75056, USA; chayyalavarthi@gmail.com (C.Y.); pooja@testgo.com (P.S.); 3 Yoda Diagnostics Pvt Ltd., Greenlands, Ameerpet, Hyderabad 500016, Telangana, India; rahulgaddipati@gmail.com

**Keywords:** gestational diabetes mellitus, hyperglycaemic pregnancy, label-free proteomics, trophoblast, BeWo cells, mitochondrial fission factor, candidate biomarker, mitochondrial dynamics, streptozotocin

## Abstract

Placental protein markers of maternal hyperglycaemia in gestational diabetes mellitus (GDM) remain scarce. We aimed to find trophoblast proteins that change under high glucose and to test whether a lead candidate is also raised in maternal serum. In our established insulin-resistant model, we used label-free liquid chromatography–tandem mass spectrometry (LC-MS/MS) to compare BeWo cells syncytialised with forskolin/8-Br-cAMP in high-glucose (25 mM) medium, with or without vascular endothelial growth factor (VEGF), with undifferentiated control cells in normoglycaemic (5 mM) medium (*n* = 3 per group); glucose and differentiation therefore varied together. Of 3528 protein groups, 388 were differentially abundant (24 at Benjamini–Hochberg q < 0.05). Mitochondrial fission factor (MFF), the outer-membrane receptor for dynamin-related protein 1 (DRP1), increased 4.5-fold (q = 0.040), whereas DRP1 was unchanged; mitochondrial calcium-uniporter components, pyruvate dehydrogenase kinase 3 and glycogen synthase 1 also increased. VEGF did not alter this signature (MFF: 0.91-fold, *p* = 0.71). Enzyme-linked immunosorbent assay (ELISA) confirmed higher MFF in high-glucose cell lysates (+48%) and, independently of in vitro differentiation, in gestational-day-7 serum of streptozotocin-treated hyperglycaemic pregnant rats (+41%; both *p* < 0.001; *n* = 3 per group), consistent with reported increases of MFF in GDM placentae. As serum was sampled after hyperglycaemia developed, these data show association, not prediction. MFF is a candidate serum biomarker of hyperglycaemic pregnancy; studies separating glucose from differentiation and validation in women are needed.

## 1. Introduction

Gestational diabetes mellitus (GDM) is a glucose-intolerance condition first recognised in pregnancy, arising when the physiological insulin resistance of gestation is not matched by adequate insulin secretion [1,2], and it is the most common metabolic complication of gestation, affecting approximately 14% of pregnancies worldwide [3,4]. Its consequences extend well beyond delivery: GDM increases the risk of pre-eclampsia, caesarean delivery, macrodsomia and neonatal hypoglycaemia, and predisposes both mother and offspring to obesity, type 2 diabetes and cardiovascular disease later in life [4,5]. Because current diagnosis relies on an oral glucose tolerance test performed at 24 to 28 weeks, a test of limited reproducibility and little first-trimester predictive value [6], there is a well-recognised need for molecular markers that report the early placental response to hyperglycaemia [7,8].

Several maternal characteristics raise the risk of GDM. Excess body weight is the most important modifiable one: in a meta-analysis of 20 cohort studies, the odds of GDM were about 2-fold higher in overweight, 3.6-fold higher in obese and 8.6-fold higher in severely obese women than in women of normal weight [9]. Diet also contributes. Prospective studies link a higher share of energy from fat at the expense of carbohydrate, and high intakes of cholesterol, haem iron and red or processed meat, to greater risk, whereas a pattern rich in fruit, vegetables, whole grains and fish appears protective [10]. Advanced maternal age, a family history of type 2 diabetes, previous GDM, polycystic ovary syndrome and ethnicity complete the recognised risk profile [4,11]. Obesity and an energy-dense diet act largely by amplifying the insulin resistance of late pregnancy [1], but maternal obesity also changes placental structure and inflammation in its own right [12], so in these pregnancies the placenta faces several overlapping metabolic stresses.

The placenta, and specifically the syncytiotrophoblast at the maternal–fetal interface, coordinates many of the metabolic adaptations of pregnancy and is a principal site of GDM pathology. Hyperglycaemia, often together with maternal obesity, increases placental oxidative stress and inflammation, perturbs vascularisation and nutrient transport, and remodels hormone and lipid handling [11,12,13,14,15]. At the organelle level, a growing literature implicates mitochondrial dysfunction and altered mitochondrial dynamics, the balance of fusion and fission, in insulin resistance and hyperglycaemic tissue stress [16,17,18,19]. Mitochondrial fission factor (MFF), a tail-anchored outer-membrane receptor that recruits the fission GTPase DRP1, is a central effector of this balance [20,21,22,23] and is itself regulated by the energy sensor AMPK under metabolic stress [24]; excessive fission and fragmentation accompany nutrient overload and are associated with reactive-oxygen-species production and insulin resistance in metabolic tissues [17,25]. In the human GDM placenta, mitochondrial dysfunction is present in both cytotrophoblast and syncytiotrophoblast mitochondria [26], and at term placental mitochondrial dynamics have been reported to shift toward fusion, possibly as an adaptation to maternal and fetal hyperinsulinaemia [27], whereas MFF protein was recently reported to be higher in GDM placentae and to rise with high glucose in extravillous trophoblast cells [28]; how the trophoblast mitochondrial machinery responds to glucose excess itself, separated from these in vivo influences, therefore remains open. The syncytiotrophoblast also continuously releases membrane-bound material, including syncytial knots, microvesicles and exosomes that carry organellar proteins, into the maternal circulation, and the abundance and cargo of these placenta-derived vesicles are altered in GDM [29,30,31]. A placental mitochondrial protein that changes with hyperglycaemia may therefore become measurable in maternal blood. Whether an unbiased, proteome-wide screen of hyperglycaemic trophoblasts identifies such changes, and whether they yield accessible circulating biomarkers, is largely unexplored.

Progress has been limited by the scarcity of tractable, well-characterised trophoblast models; primary trophoblasts are heterogeneous and short-lived [32]. We previously established and validated an insulin-resistant, glucose-intolerant BeWo model in which cells syncytialised with forskolin/8-Br-cAMP in 25 mM glucose medium show a significant reduction in insulin-stimulated 2-NBDG uptake by flow cytometry relative to undifferentiated cells in normoglycaemic medium, and in which exogenous VEGF does not significantly restore glucose uptake [33]. That study described the functional defect but not the protein changes behind it.

Here, we applied label-free quantitative proteomics to this model. The approach has been used to survey placental tissue, circulating exosomes and maternal plasma in GDM [7,8,34,35,36,37,38,39], but not, to our knowledge, a trophoblast model with a defined insulin-resistant phenotype. We compared BeWo cells syncytialised in high-glucose medium with undifferentiated normoglycaemic cells, as in our established model [33], and included a VEGF-treated arm because exogenous VEGF had not restored glucose uptake in that model [33]. Differential abundance was tested with moderated statistics and false-discovery-rate control [40,41], and enrichment was assessed against the Gene Ontology and KEGG databases [42,43,44]. We then looked closely at the fission–fusion, mitochondrial-calcium and carbon-flux proteins quantified in the same dataset, because changes in mitochondrial dynamics accompany insulin resistance in other metabolic tissues [16,17,18]. The lead protein, MFF, was measured by sandwich ELISA in cell lysates and in serum of streptozotocin (STZ)-treated pregnant rats, a widely used model of hyperglycaemic pregnancy [45,46,47]. Because serum was collected after hyperglycaemia had been established, this design tests whether MFF is associated with hyperglycaemic pregnancy, which would make it a candidate diagnostic or monitoring signal, and not whether it predicts GDM before onset [48].

The study therefore had three aims: (i) to define how the proteome of the high-glucose, insulin-resistant BeWo model differs from that of normoglycaemic control cells, and whether VEGF modifies this difference; (ii) to identify among the changed proteins a mitochondrial candidate that reports trophoblast exposure to high glucose; and (iii) to test whether this candidate is also raised in maternal serum in an in vivo model of hyperglycaemic pregnancy. We hypothesised that high glucose shifts the abundance of trophoblast mitochondrial-dynamics proteins and that at least one such change can be detected in the maternal circulation. To our knowledge, this is the first trophoblast proteome of high-glucose exposure to be carried through to serum-level confirmation of a specific candidate in vivo [37,39,49].

## 2. Materials and Methods

### 2.1. Cell Model, Culture Conditions and Experimental Timeline

**Figure 1:**
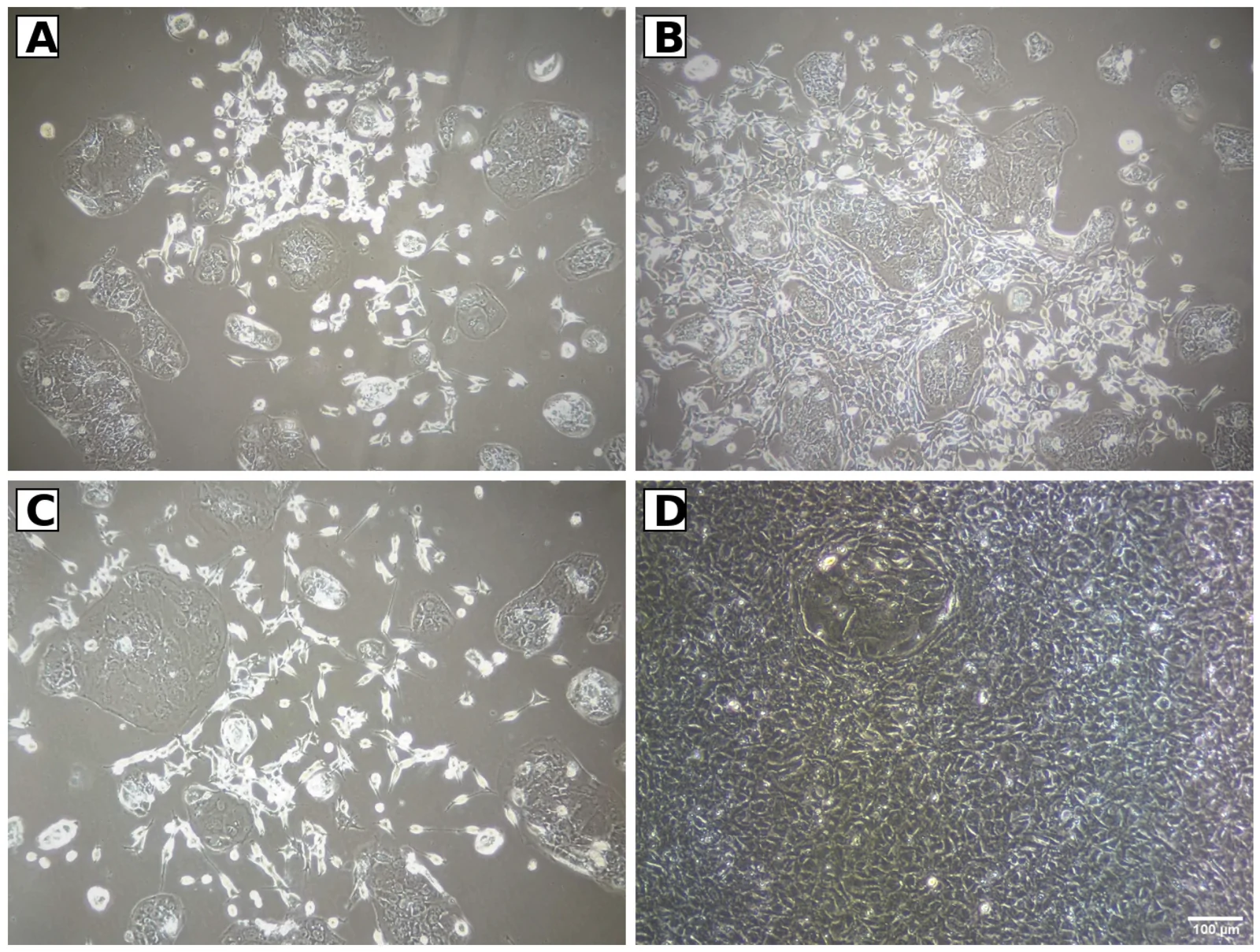
Phase-contrast micrographs of the BeWo trophoblast model. (**A**–**C**) Undifferentiated BeWo cells at increasing confluency in LG medium, showing epithelial colony morphology, representative of the Control condition. (**D**) After treatment with 40 µM forskolin and 250 µM 8-Br-cAMP in HG medium, cells form a confluent multinucleated syncytial sheet (scale bar 100 µm), representative of the Test condition. Cells are representative of the model characterised in [33]. The panels show cell-level syncytialisation (plasma-membrane fusion of trophoblasts); mitochondrial morphology, clustering or fusion was not imaged.

The insulin-resistant BeWo trophoblast model was generated and phenotypically characterised as reported [33], and the cultures profiled here followed the same protocol. BeWo human choriocarcinoma trophoblast cells were obtained from the national cell repository at the National Centre for Cell Science (NCCS, Pune, India), revived from the repository stock and used after three passages, as in [33]; STR re-authentication and mycoplasma testing were not documented for these cultures (see Limitations). Two media were used (Ham’s F-12K, MP Biomedicals, Illkirch, France): LG (normoglycaemic) medium, Ham’s F-12K at 5 mM D-glucose, supplemented with 10% fetal bovine serum (FBS; HiMedia Laboratories, Mumbai, India); and HG medium, Ham’s F-12K supplemented with D-glucose (Sigma-Aldrich/Merck KGaA, Darmstadt, Germany) to 25 mM and with 2% FBS [33]; both media contained 1% penicillin–streptomycin (HiMedia Laboratories), and cultures were maintained at 37 °C in a humidified 5% CO_2_ atmosphere. For each biological replicate, 5 × 10^5^ cells were seeded in a T-25 flask (BioLite; Thermo Fisher Scientific, Waltham, MA, USA) in LG medium and allowed to attach overnight (day 0). On day 1, the medium of the Test and Test+VEGF flasks was removed, the cells were rinsed with DPBS (HiMedia Laboratories), and HG medium containing 40 µM forskolin and 250 µM 8-Br-cAMP (both Sigma-Aldrich) was added; the treatment was renewed on day 3. Syncytialisation and high-glucose exposure therefore proceeded together, and these cultures formed multinucleated syncytial sheets (Figure 1D) with reduced insulin-stimulated 2-NBDG uptake [33]. The Control flasks remained in LG medium without forskolin or 8-Br-cAMP (Figure 1A–C). Cell morphology was documented by phase-contrast microscopy (XDFL-series inverted microscope; SDPTOP, Sunny Instruments, Ningbo, China), and images were processed in ImageJ/Fiji 1.53c (National Institutes of Health, Bethesda, MD, USA). On day 3, 50 ng/mL recombinant human VEGF_165_ (HEK 293-expressed, Cat. No. Z03073; GenScript, Piscataway, NJ, USA) was added to the Test+VEGF flasks, and all flasks were incubated for a further 18 h before harvest, giving ~72 h of HG exposure for the Test groups, comparable to previous BeWo hyperglycaemia studies [50]. Control and Test therefore differ in three respects: glucose (5 vs. 25 mM), forskolin/8-Br-cAMP-induced syncytialisation (absent vs. present) and serum (10% vs. 2% FBS). This design reproduces the insulin-resistant model of [33] but does not allow the effect of glucose to be separated from that of differentiation or serum reduction; the comparison is therefore interpreted as the high-glucose differentiated state versus undifferentiated normoglycaemic cells (see Discussion and Limitations). The Test+VEGF arm shares the Control group, so the in vitro design is a three-group (not factorial) design. The additional 20 mM glucose raises the calculated osmolarity of HG medium by ~20 mOsm/L (≈7%); osmolarity was not measured, and no iso-osmolar mannitol control was included. Three biological replicates per group (independent flasks processed separately) were profiled, each by a single LC-MS/MS injection (no technical replicates). Viability of the model was >96% by trypan-blue exclusion in our previous characterisation [33] but was not measured separately in each group of the present cultures; because equal amounts of protein were digested and injected (Section 2.2), the proteomic data are normalised to total protein rather than to cell number. At harvest, monolayers were washed twice with ice-cold PBS, scraped, pelleted (500× *g*, 5 min, 4 °C) and snap-frozen. Throughout this paper, ‘Test’, ‘high glucose’ and ‘hyperglycaemic’ in the in vitro context refer to this combined high-glucose differentiated condition.

### 2.2. Protein Extraction, Sample Preparation and LC-MS/MS

Cell pellets were lysed in RIPA buffer supplemented with a protease-inhibitor cocktail, clarified by centrifugation (12,000× *g*, 15 min, 4 °C) and quantified by bicinchoninic-acid assay. Next, 100 µg protein per sample was reduced (5 mM TCEP), alkylated (50 mM iodoacetamide) and digested with trypsin (1:50, 16 h, 37 °C). Peptides were desalted (C18), vacuum-dried and reconstituted in 2% acetonitrile/0.1% formic acid. Then, 1 µg peptide was separated on a 15 cm Acclaim PepMap C18 column (3 µm; Thermo Fisher Scientific, Waltham, MA, USA) over a 0–40% acetonitrile gradient at 500 nL/min (Easy-nLC 1000; Thermo Fisher Scientific) coupled to an Orbitrap Exploris mass spectrometer (Thermo Fisher Scientific; 60 min runs). MS1: R = 60,000, *m*/*z* 375–1500, AGC 300%, max IT 25 ms, dynamic exclusion 30 s; MS2: top-12 DDA, R = 15,000, AGC 200%, max IT 22 ms. Nine runs (3 groups × 3 replicates) were acquired.

### 2.3. Database Search and Label-Free Quantification

RAW files were processed in Proteome Discoverer 2.5 (Thermo Fisher Scientific, Waltham, MA, USA) with dual Sequest HT and MS Amanda 2.0 searches against UniProt human (precursor 10 ppm, fragment 0.02 Da; trypsin/P; fixed carbamidomethyl-Cys; variable Met oxidation, N-terminal acetylation). PSM and protein-group FDR were controlled at ≤1% [51,52]. Label-free quantification used precursor intensities. Protein-group abundances were log_2_-transformed and normalised between samples so that every sample had the same mean log_2_ abundance. For each pairwise comparison, proteins quantified in at least two of the three replicates of both groups were retained (3271 proteins for Test vs. Control and 3260 for Test+VEGF vs. Control), and the remaining missing values (at most one per group) were imputed by the processing facility with low-abundance values (approximately 4.7 log_2_ units below the sample mean). These normalised log_2_ abundances are the values that were tested statistically and are presented in Section 3.4.

### 2.4. Statistical and Bioinformatic Analysis

Differential abundance between two groups was assessed on the normalised log_2_ abundances with the empirical-Bayes moderated *t*-test of the limma framework [40]; the reported *p*-values were reproduced exactly from the normalised data with this model. Proteins with *p* < 0.05 were considered differentially abundant (DAPs), and the Benjamini–Hochberg procedure [41] was applied to control the false-discovery rate, reporting counts at q < 0.05 and q < 0.10. Effects are estimated as log_2_ fold changes (difference of group mean log_2_ abundances) and are reported in the tables as linear fold changes (FC = 2^log2FC^; FC < 1 indicates a decrease). z-Scores (each protein centred and scaled across samples) were used only for visualisation and sample-level quality control: (i) pairwise Pearson correlation, summarised as median within- and between-group coefficients; (ii) principal-component analysis (PCA) by singular-value decomposition; and (iii) Ward-linkage hierarchical clustering on Euclidean distance. Because PCA and clustering restricted to DAPs are partly circular, both were repeated without feature selection on all 3271 quantified proteins. Concordance between the two pairwise comparisons (Test vs. Control and Test+VEGF vs. Control) was quantified by the overlap of DAP sets and the Jaccard index. To analyse the complete in vitro design with prespecified contrasts, all nine samples were additionally re-analysed in a single three-group linear model (Control, Test, Test+VEGF) on median-normalised log_2_ abundances without imputation (proteins retained when quantified in at least two replicates of both groups of a contrast), with a pooled residual variance, empirical-Bayes moderation and three prespecified contrasts, Test vs. Control, Test+VEGF vs. Control and Test+VEGF vs. Test, each corrected by the Benjamini–Hochberg procedure (Table S7). Because no Control+VEGF arm was included, a factorial (glucose × VEGF) model could not be fitted. Functional over-representation of the up- and down-regulated sets was tested separately against the human background by the modified Fisher exact test (EASE) with Benjamini–Hochberg correction for KEGG pathways and GO Biological Process, Molecular Function and Cellular Component terms [42,43,44]. A targeted, hypothesis-generating interrogation of the same dataset was then performed: proteins of the mitochondrial fission–fusion machinery (MFF, DNM1L/DRP1, FIS1, OPA1), mitochondrial calcium handling (MCU, MICU1, VDAC1, PPP3R1, CALM3), pyruvate-dehydrogenase regulation and carbon storage (PDK3, PDHA1, PDHB, PDP1, GYS1, ACADVL, NPC1), oxidative phosphorylation and mitochondrial stress (ATP5PF, ATP5F1B, NDUFAB1, SOD2, BNIP1) and canonical insulin signalling (AKT1, AKT2, GSK3B, SLC2A1, PRKAA1) were extracted with their fold changes and *p*-values in all comparisons. To assess a possible osmotic contribution, canonical hypertonicity- and stress-responsive proteins quantified in the dataset (AKR1B1, AQP1, HSPA5, HSPA8, HSPH1, DNAJB1, HMOX1) were examined in the same way (Table S9). Co-abundance of MFF with every other protein quantified in all eight samples in which MFF was detected was assessed by Pearson correlation of log_2_ abundances. All analyses were performed in Python 3.13 (NumPy 2.5.3, SciPy 1.18.1) and R 4.2.1 (R Foundation for Statistical Computing, Vienna, Austria); a two-sided *p* < 0.05 was considered significant.

### 2.5. Streptozotocin-Induced Hyperglycaemic Pregnant-Rat Model

All animal procedures were approved by the Institutional Animal Ethics Committee of Lovely Professional University (LPU/IAEC/2022/17; approved 3 October 2022), conducted under CPCSEA (Government of India) guidelines and reported in accordance with ARRIVE 2.0 [53]. Sixteen healthy adult Sprague-Dawley rats (12 nulliparous adult females, body weight 275–390 g before mating, and 4 proven-breeder males) from the Central Animal House Facility, Lovely Institute of Technology (Pharmacy), were acclimatised for 7 days in polypropylene cages under identical controlled conditions (22 ± 2 °C, 55 ± 10% relative humidity, 12 h light/dark cycle) with a standard normocaloric laboratory rodent pellet diet and water ad libitum. The same diet was fed throughout mating and gestation; no high-fat, high-sugar or otherwise modified diet was used, so hyperglycaemia in this model resulted from STZ alone. Females were co-housed overnight with males (3:1) and pregnancy was confirmed the following morning by the presence of a vaginal plug or spermatozoa in the vaginal smear; that day was designated gestational day (GD) 0. Pregnant females were randomly allocated to two groups (*n* = 6 each; Table 1). Body weight of the STZ-allocated dams was 325.8 ± 47.1 g (range 275–390 g) before mating and 393.3 ± 54.6 g (range 330–468 g) at GD0. Hyperglycaemia was induced by intraperitoneal injection of freshly prepared streptozotocin (Sigma-Aldrich; 35 mg/kg body weight in ice-cold 0.1 M sodium-citrate buffer, pH 4.5) once daily for five consecutive days (GD1–GD5) [45,46]; controls received an equal volume of vehicle on the same schedule. This multiple-low-dose regimen was chosen to produce a stable, moderate gestational hyperglycaemia rather than the fulminant β-cell ablation caused by a single high dose [46]; it reproduces maternal hyperglycaemia through β-cell injury and insulin deficiency, not the insulin resistance of human GDM [45,47], and is therefore described here as a model of hyperglycaemic pregnancy. Fasting (6 h) blood glucose was measured from tail-vein blood with a calibrated glucometer (CareSens II Plus; i-SENS, Seoul, Republic of Korea) at baseline (GD0, before STZ) and on GD7 (48 h after the last STZ dose); a fasting glucose > 200 mg/dL defined diabetes [46], and every STZ-treated animal met this criterion. Body weight was recorded before mating (non-pregnant baseline), at GD0 (before STZ) and on GD7. On GD7 (early gestation in the rat, term ≈ GD21–22), blood (~0.5 mL) was collected from the retro-orbital sinus under light isoflurane anaesthesia, allowed to clot for 30 min at room temperature and centrifuged (3000× *g*, 15 min, 4 °C); serum was aliquoted and stored at −80 °C until MFF ELISA. Because STZ had been given on GD1–GD5, hyperglycaemia was already established at sampling, so this design tests association with hyperglycaemic pregnancy rather than prediction. The MFF ELISA was planned as a pilot-scale validation: serum from three dams per group was selected before the assay and analysed, matching the triplicate design of the in vitro experiments; all other in vivo analyses used all six STZ-treated dams. Fasting glucose and body weight were recorded for the STZ-allocated dams; the study records contain no corresponding values for the vehicle-treated dams, so glucose and body weight are analysed as within-animal comparisons in the STZ group (each dam serving as its own control), and the vehicle group serves as the comparator for serum MFF. Glucose was compared by paired *t*-test, and body weight across the three time points by repeated-measures one-way ANOVA (Greenhouse–Geisser-corrected) with Šidák-adjusted paired comparisons. The individual dam was the experimental unit for all in vivo analyses; fetuses and placentae were not analysed, so litter effects do not arise. Investigators were not blinded to treatment during induction and sampling; glucose, body-weight and ELISA read-outs were instrument-based. Animals were monitored daily for welfare, and no animal met humane-endpoint criteria or was excluded from analysis.

### 2.6. Sandwich-ELISA Quantification of MFF in Cell Lysate and Serum

Rationale for serum measurement. MFF is a tail-anchored outer-mitochondrial-membrane protein [20,21]. Because the syncytiotrophoblast sheds membrane-bound vesicles and cellular material carrying organellar proteins into the maternal circulation, and because the quantity and protein cargo of these placenta-derived vesicles change in GDM [29,30,31], a high-glucose-associated increase in trophoblast MFF could be detectable as a rise in circulating MFF; hyperglycaemia additionally promotes mitochondrial fission in maternal insulin-target tissues [17,18], so serum represents an integrated, clinically accessible read-out. Assay. MFF was quantified with a commercial two-site sandwich ELISA (Human Mitochondrial Fission Factor ELISA Kit, Abbkine Scientific Co., Ltd., Wuhan, China; Cat. No. KTE61641; Lot No. ATXD08082; 96 tests; species reactivity: human) according to the manufacturer’s instructions (stated range 25–400 pg/mL; minimum detectable dose < 1 pg/mL; intra-/inter-assay CV < 9%/<11%) in four sample types: BeWo cell lysate from Control and Test cultures (*n* = 3 independent cultures per group, loaded at equal total protein) and GD7 serum from vehicle-treated and STZ-treated pregnant rats (*n* = 3 dams per group; Section 2.5). Each sample was assayed in replicate wells, and the mean absorbance of the technical replicates was interpolated, so that each biological replicate (culture or dam) contributed one value to the analysis; all samples were run on one plate together with the kit calibrator series and blanks, so no inter-plate correction was required. The kit is calibrated with human MFF. Cross-reactivity with rat MFF is supported by the conservation of MFF between the two species and by the finding that all rat-serum absorbances fell within the calibrated range and on the same segment of the curve as the cell lysates; however, rat-matrix validation (spike-recovery and dilution linearity in rat serum) was not performed. Serum values are therefore reported as relative, kit-specific MFF immunoreactivity, suitable for the within-assay comparison of vehicle and STZ dams, and not as absolute rat MFF concentrations. Absorbance was read at 450 nm on a microplate reader (BK-EL10A; Biobase, Jinan, China) and interpolated against a four-parameter-logistic (4PL) calibration curve fitted to the calibrators in GraphPad Prism 9.5.1 (GraphPad Software, Boston, MA, USA); fitted parameters: Bottom = 0.145, Top = 2.991, EC_50_ = 40.5 pg/mL, Hill slope = 2.25; R^2^ = 0.9993; replicates test for lack of fit, *p* = 0.91. All twelve sample absorbances (1.69–2.41) lay on the steep, most sensitive segment of the curve between the EC_50_ and ~80% of the upper asymptote, where interpolation error is minimal (Section 3.9). The primary analysis compared the four groups by ordinary one-way ANOVA followed by Šidák’s multiple-comparisons test (α = 0.05); equality of variances was confirmed by the Brown–Forsythe test (F(3,8) = 0.56, *p* = 0.66) and Bartlett’s test (χ^2^(3) = 3.79, *p* = 0.29). Effects are reported as mean differences with Šidák-adjusted 95% confidence intervals; because cell lysate and serum are different matrices, each matrix was also analysed separately by Welch’s two-sample *t*-test as a sensitivity analysis (Section 3.9).

## 3. Results

### 3.1. The Syncytialised BeWo Trophoblast Model

The BeWo choriocarcinoma trophoblast model was established and phenotypically characterised as previously reported [33]. Control cultures were undifferentiated BeWo cells in LG medium, which formed characteristic epithelial colonies (Figure 1A–C). Test and Test+VEGF cultures were treated with 40 µM forskolin and 250 µM 8-Br-cAMP in HG medium and differentiated into multinucleated syncytia forming a confluent syncytiotrophoblast-like sheet (Figure 1D); in this high-glucose differentiated state, insulin-stimulated 2-NBDG uptake is reduced relative to Control cells [33]. These three states were compared by label-free proteomics. Figure 1 documents cell-level syncytialisation, i.e., fusion of trophoblast plasma membranes into multinucleated syncytia; it does not report mitochondrial morphology, and the cell aggregates visible in Figure 1A–C should not be interpreted as mitochondrial clustering or fusion.

### 3.2. A Deep, Highly Reproducible Trophoblast Proteome Separates High-Glucose from Control Cells

**Figure 2:**
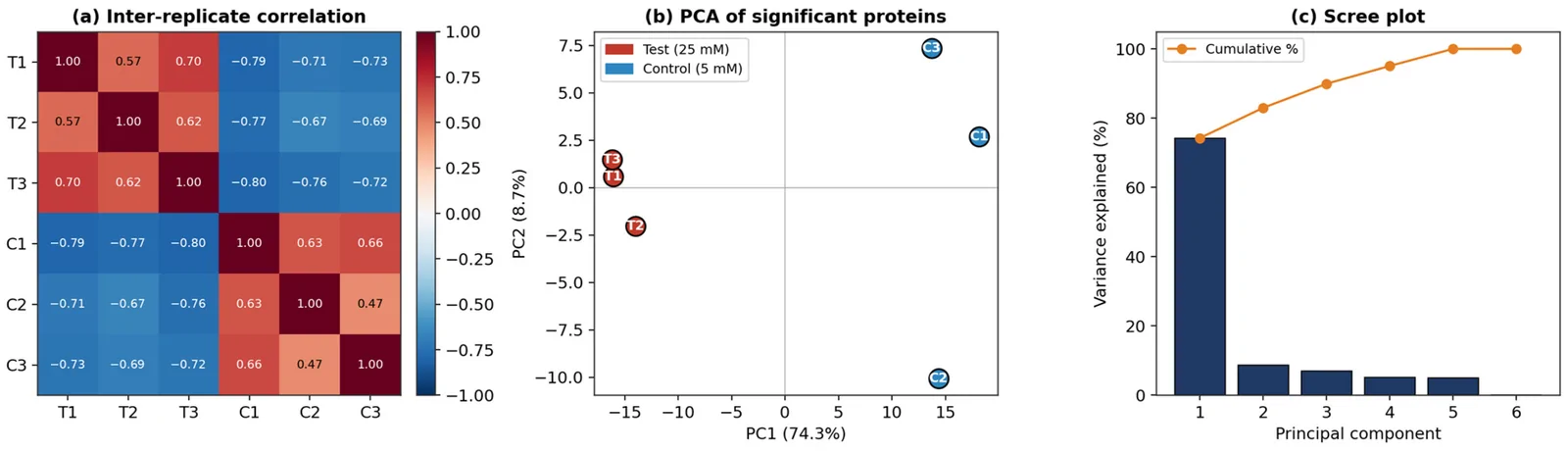
Sample-level quality control. (**a**) Pairwise Pearson-correlation matrix of z-scored abundances of the 388 DAPs (T, Test; C, Control). (**b**) PCA of the DAPs; PC1 separates the conditions and explains 74.3% of the variance of the DAP set (on all 3271 quantified proteins without feature selection, PC1 = 32.8% and also separates the groups; Table 2). (**c**) Scree plot with cumulative variance.

**Figure 3:**
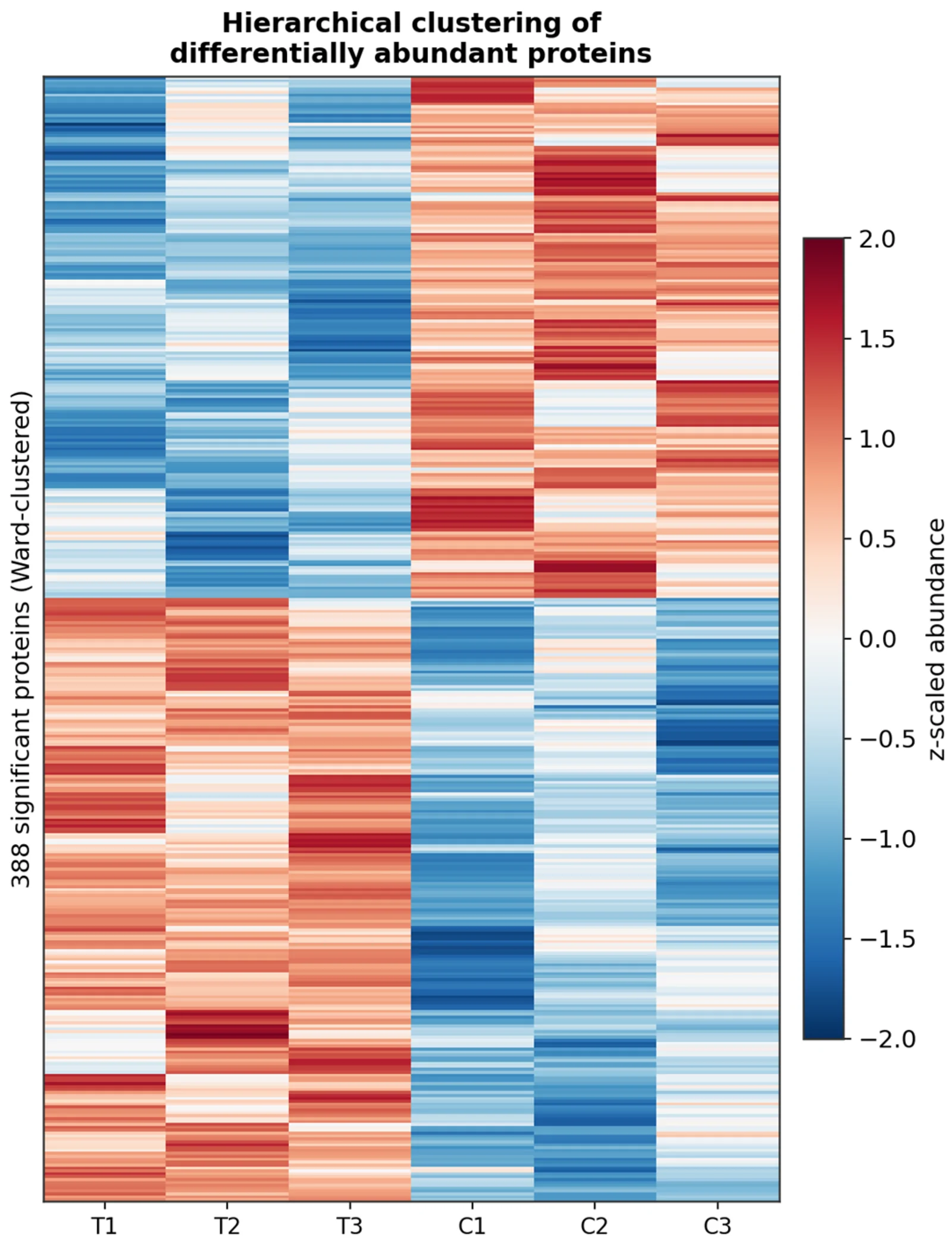
Ward-linkage hierarchical clustering of the 388 differentially abundant proteins (z-scaled abundance); the two conditions form distinct blocks.

Across nine LC-MS/MS runs we confidently identified 3528 protein groups from 22,431 peptides at ≤1% FDR (Table 2). A core of 3357 proteins (95.2%) was common to all three groups, with only 31, 15 and 31 proteins unique to Control, Test and Test+VEGF respectively, indicating that the high-glucose differentiated state re-tunes the abundance of a shared proteome rather than altering the protein complement wholesale. Quality control confirmed a reproducible condition effect. On the DAPs, replicates correlated positively within groups (median r = 0.63) while Control and Test samples were anti-correlated on z-scored abundances (median r = −0.73; Figure 2a), PCA resolved the groups along the first component (74.3% of the variance of the DAP set; PC2 = 8.7%; Figure 2b,c), and Ward clustering of the DAPs recovered the two conditions as distinct blocks (Figure 3). Because these DAP-based views are partly circular, we repeated them without feature selection on all 3271 quantified proteins: PC1 explained 32.8% of the total variance and separated every Test sample from every Control sample, and unsupervised Ward clustering again recovered the two groups exactly. The condition-associated proteomic signal is therefore present in the whole proteome and is not an artefact of feature selection or of individual replicates.

### 3.3. High Glucose Significantly Remodels the Trophoblast Proteome

**Figure 4:**
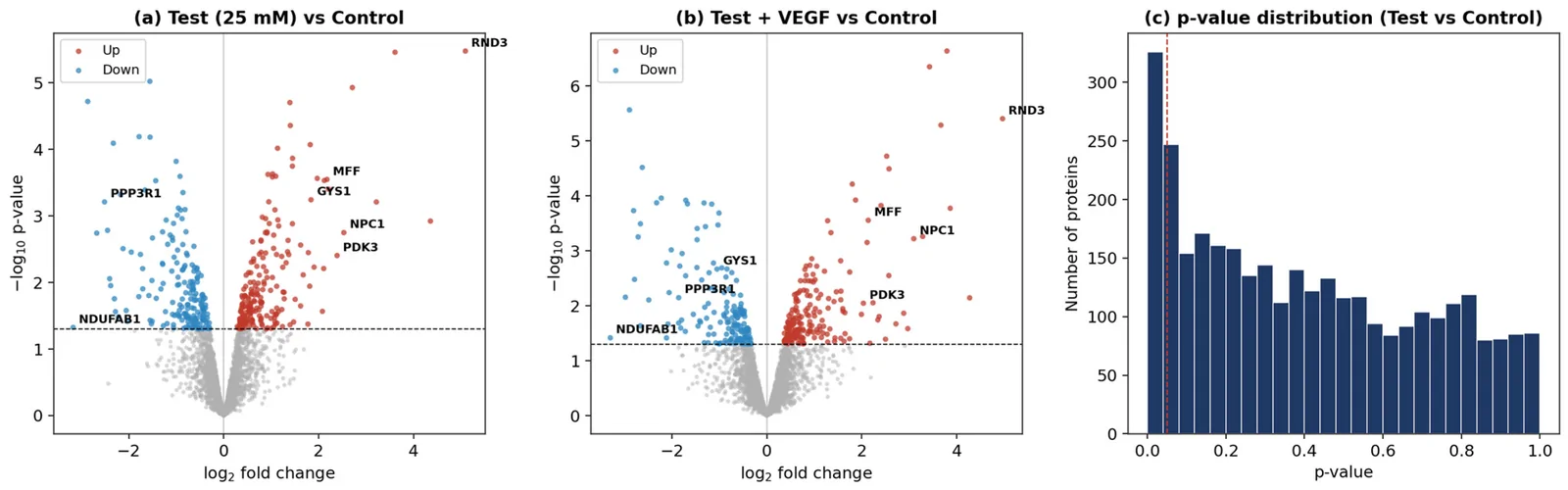
Differential abundance. Volcano plots for (**a**) Test vs. Control and (**b**) Test+VEGF vs. Control (red = up, blue = down at *p* < 0.05; black dashed line, *p* = 0.05); selected mitochondrial/metabolic proteins are labelled. (**c**) *p*-value histogram for Test vs. Control showing marked enrichment near zero (red dashed line, *p* = 0.05).

Comparing Test with Control, 388 proteins were differentially abundant at *p* < 0.05 (208 up, 180 down; Figure 4a), of which 24 remained significant after Benjamini–Hochberg FDR control at q < 0.05 and 47 at q < 0.10, a substantial signal for an *n* = 3 design. The *p*-value distribution was strongly right-skewed toward zero (Figure 4c), consistent with a large fraction of true positives rather than uniform noise. The 24 FDR-significant proteins (Table 3) were led by RhoE/RND3 (+34-fold), the SNARE VTI1B (+12-fold) and the phosphatase activator PTPA, and prominently included the mitochondrial and metabolic proteins MFF (+4.5-fold, q = 0.040), the fatty-acid-oxidation enzyme ACADVL (+2.2-fold) and the mitochondrial apoptosis regulator BNIP1 (+2.1-fold), alongside down-regulated NCK1, UTP20, CTBP2 and QDPR. The three-group re-analysis of all nine samples without imputation (Section 2.4; Table S7) was concordant: the Test-vs.-Control contrast yielded 451 nominal DAPs and 28 at q < 0.05, recovered 314 of the 388 original DAPs (log_2_ fold-change correlation r = 0.94) and retained MFF as FDR-significant (4.59-fold, q = 0.025). Because the Test cultures were also syncytialised, we looked for differentiation and proliferation markers: canonical syncytiotrophoblast markers (hCG subunits, syncytin-1 and -2, pregnancy-specific glycoproteins, CYP19A1, HSD11B2, GDF15) were below the detection limit of this LFQ dataset, and proliferation markers did not differ significantly (PCNA 0.79-fold, *p* = 0.053; MCM2, MCM3, MCM7, TOP2A and CDK1 unchanged), so the contribution of differentiation cannot be estimated directly from the proteome. Likewise, the reduction of serum in the Test medium did not produce a detectable serum-deprivation response: autophagy (SQSTM1/p62, LC3B), endoplasmic-reticulum-stress (HSPA5) and SREBP-dependent lipogenic (FASN, ACACA, SCD) proteins were unchanged (all *p* > 0.15).

### 3.4. Up-Regulated Proteins Include Mitochondrial-Dynamics and Energy-Metabolism Proteins

**Figure 5:**
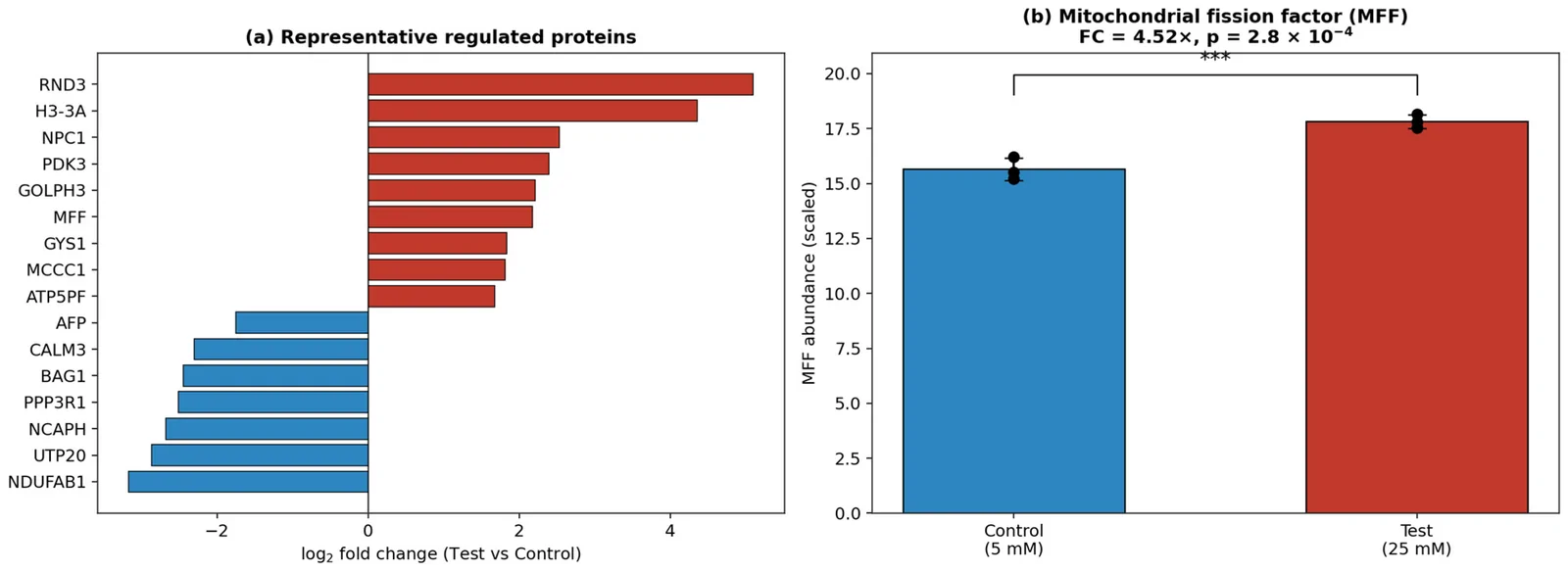
(**a**) Representative up- (red) and down-regulated (blue) proteins ranked by log_2_ fold change. (**b**) MFF normalised log_2_ abundance across replicates (bars = mean ± SD, points = replicates); *** moderated *t*-test *p* < 0.001.

Beyond the FDR-significant core, the broader up-regulated set (Table 4, Figure 5a) was dominated by mitochondrial and metabolic machinery: the fission receptor MFF; pyruvate dehydrogenase kinase 3 (PDK3, +5.2-fold), which restrains pyruvate oxidation; glycogen synthase (GYS1, +3.6-fold); the branched-chain enzyme MCCC1; the cholesterol transporter NPC1 (+5.8-fold); and a coordinated rise in mitochondrial solute carriers (SLC25A3/4/11/20) and ATP-synthase subunits (ATP5PF, ATP5F1B, ATP5ME). Conversely, the respiratory-chain/acyl-carrier subunit NDUFAB1 (−9-fold), the calcineurin regulatory subunit PPP3R1 and calmodulin CALM3, which are nodes of calcium/insulin signalling, and alpha-fetoprotein (AFP) were down-regulated (Figure 5a). The abundance of MFF was tightly separated between conditions (normalised log_2_ abundance, Control 15.65 ± 0.51 vs. Test 17.83 ± 0.31; Figure 5b).

### 3.5. Enrichment Analysis Recovers Mitochondrial and Insulin-Resistance-Related Annotations

**Figure 6:**
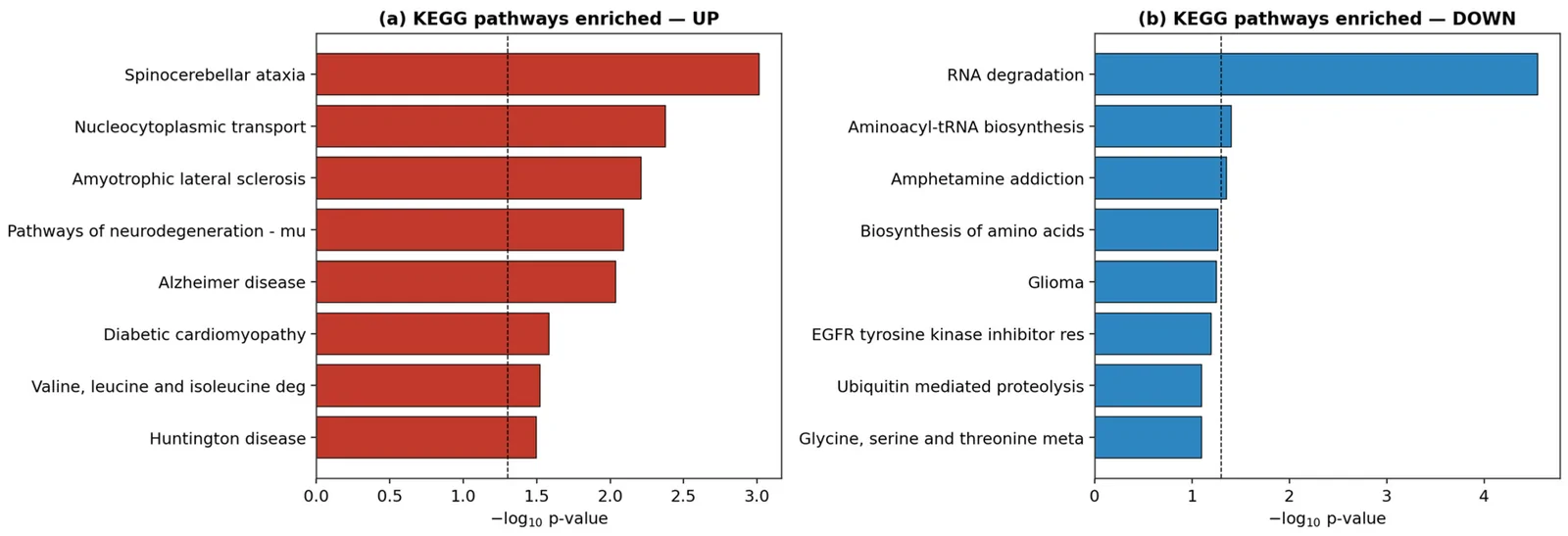
KEGG pathway enrichment for (**a**) up- and (**b**) down-regulated proteins (bars = −log10 *p*; dashed line *p* = 0.05).

**Figure 7:**
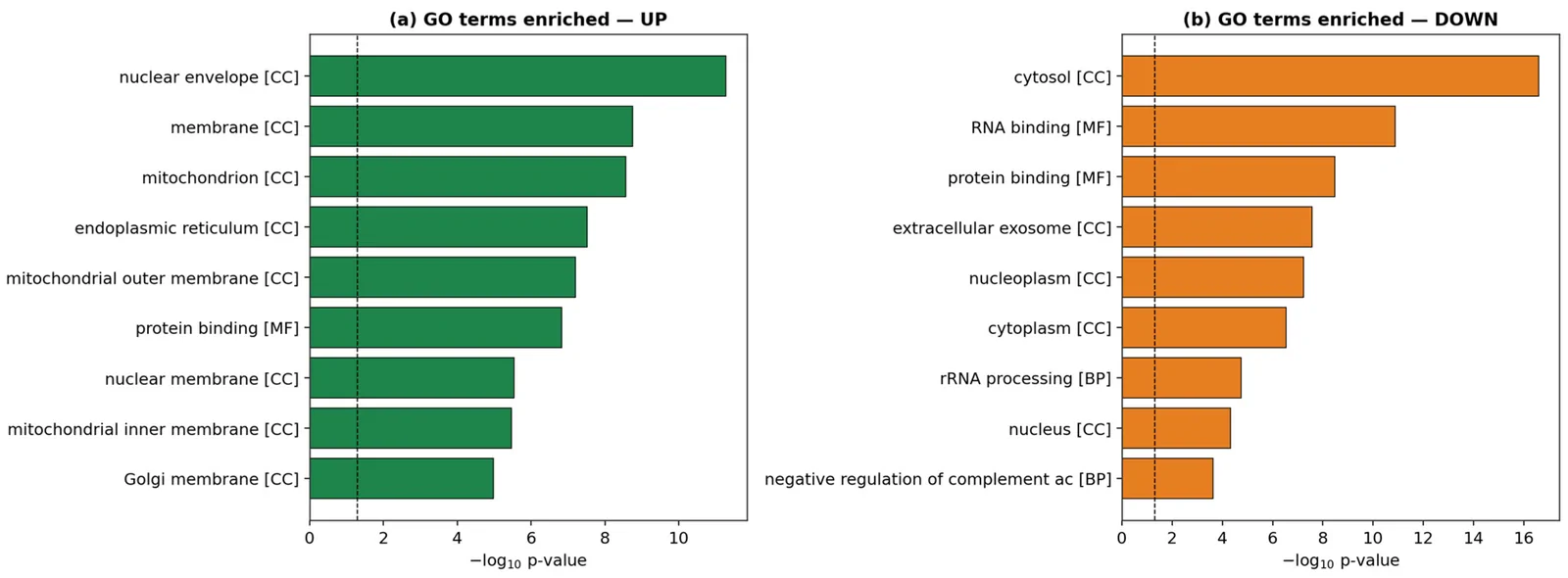
Gene Ontology enrichment (BP/CC/MF combined) for (**a**) up- and (**b**) down-regulated proteins.

KEGG over-representation of the up-regulated set recovered insulin resistance, diabetic cardiomyopathy, cholesterol metabolism, glycerolipid metabolism and branched-chain amino-acid degradation at nominal significance, a metabolically coherent portrait of the high-glucose trophoblast (Table 5, Figure 6a). The down-regulated set was dominated by RNA degradation, which was the only term to survive FDR correction (BH-FDR = 6.3 × 10^−3^), together with aminoacyl-tRNA and amino-acid biosynthesis, indicating a coordinated dampening of protein-synthesis capacity (Figure 6b). Gene Ontology reinforced this (Table 6, Figure 7): up-regulated proteins were enriched for mitochondrion and the mitochondrial outer membrane (both significant after FDR correction), endoplasmic reticulum and positive regulation of cell growth, whereas down-regulated proteins were enriched for RNA binding, rRNA processing and translational initiation. The smaller biological-process terms ‘calcium import into the mitochondrion’ (ITPR1, AFG3L2, VDAC1, MICU1) and ‘mitochondrial fusion’ (OPA1, MFF, AFG3L2, CHCHD3) were nominally enriched but did not survive FDR correction (q = 0.11 and 0.27). These terms are annotation-based and driven by four proteins each, one of which (MFF) is itself annotated to ‘mitochondrial fusion’; they indicate that mitochondrial-dynamics and calcium-handling proteins are over-represented among the changed proteins, but they do not establish that mitochondrial fusion, fission or calcium import is increased. Enrichment and differential abundance are also not statistically independent, because enrichment is computed on the DAP list; we therefore regard them as complementary views of the same signal.

### 3.6. Targeted Interrogation of Mitochondrial-Dynamics, Calcium-Handling and Carbon-Flux Proteins Generates a Testable Hypothesis Centred on MFF

To move beyond enrichment statistics, we extracted from the same quantitative dataset every quantified component of the mitochondrial fission–fusion machinery, the mitochondrial calcium-uniporter complex, the pyruvate-dehydrogenase (PDH) regulatory node and canonical insulin signalling, and asked whether their abundance changes were internally consistent (Table 7). Three features emerged; all are changes in protein abundance, and none was tested functionally. First, among fission proteins the change was selective for the receptor: MFF rose 4.5-fold (q = 0.040), whereas the fission GTPase it recruits, DNM1L/DRP1 (0.84-fold, *p* = 0.13), and the alternative DRP1 receptor FIS1 (1.12-fold, *p* = 0.31) were unchanged, and the inner-membrane fusion and cristae-organising GTPase OPA1 rose modestly (1.54-fold, *p* = 0.027). Because MFF is the principal outer-membrane receptor for DRP1 [23], a selective rise in MFF with constant DRP1 is compatible with an increased capacity for DRP1 recruitment. Abundance alone, however, does not establish that fission is increased, that MFF is rate-limiting, or that the modest OPA1 change increases fusion: DRP1 recruitment and phosphorylation, mitochondrial morphology and fusion–fission rates were not measured, and the outer-membrane fusion proteins MFN1 and MFN2 were not quantified. The concurrent increase in MFF and OPA1 is equally consistent with a remodelled, rather than a uni-directionally shifted, mitochondrial network. Second, the mitochondrial calcium-import machinery was co-increased: MCU (2.54-fold, *p* = 0.075), its regulator MICU1 (1.80-fold, *p* = 0.0098) and the outer-membrane channel VDAC1 (1.22-fold, *p* = 0.046), while the cytosolic calcium effectors calcineurin-B (PPP3R1, −5.7-fold, *p* = 6.2 × 10^−4^) and calmodulin-3 (CALM3, −4.9-fold, *p* = 0.018) decreased; this pattern is compatible with, but does not demonstrate, a shift of calcium handling toward the mitochondria [54]. Third, among PDH regulators PDK3 rose 5.2-fold (*p* = 0.0039) while the PDH catalytic subunits PDHA1 and PDHB and the PDH phosphatase PDP1 were unchanged, which predicts, but does not demonstrate, kinase-mediated restraint of pyruvate oxidation [55]; concomitantly, glycogen synthase (GYS1, +3.6-fold), the β-oxidation enzyme ACADVL (+2.2-fold) and the cholesterol transporter NPC1 (+5.8-fold) increased. In contrast, the canonical insulin-signalling proteins AKT1, AKT2, GSK3B, GLUT1 (SLC2A1) and the AMPK catalytic subunit PRKAA1 were unchanged in abundance; their phosphorylation state was not assessed.

Two further observations place MFF at the centre of this signature. Across the eight samples in which MFF was quantified, its log_2_ abundance correlated most strongly with MICU1 (r = 0.95, *p* = 3.4 × 10^−4^) and BNIP1 (r = 0.91, *p* = 1.8 × 10^−3^), and inversely with the mitochondrial single-stranded-DNA-binding protein SSBP1 (r = −0.91, *p* = 1.9 × 10^−3^); these are correlations across samples that largely reflect the shared condition effect; like co-expression analyses of placental transcriptomes [56], they identify proteins that change together but do not imply physical or functional interaction. The axis also behaved concordantly in the second set of high-glucose cultures that additionally received VEGF (Table 7, Test+VEGF columns): MFF (4.39-fold, *p* = 2.8 × 10^−4^; quantified in two of the three Test+VEGF cultures, the third value being imputed), PDK3 (4.09-fold), MCU (2.66-fold), MICU1 (2.12-fold), ACADVL (2.55-fold), BNIP1 (2.07-fold) and NPC1 (8.55-fold) were again increased; DRP1, FIS1, PDHA1/PDHB and the insulin-signalling proteins were again unchanged; and PPP3R1, CALM3 and NDUFAB1 were again decreased. Because the Test+VEGF cultures share the Control group, this is a concordance check rather than a fully independent replication; the direct Test+VEGF-vs.-Test contrast is reported in Section 3.7. The one exception, GYS1, which was reduced rather than induced in the presence of VEGF (0.48-fold vs. Control, *p* = 0.0022), identifies glycogen synthase as the single VEGF-responsive node in the axis. We summarise these observations as a hypothesis (Section 3.10): the high-glucose differentiated state is associated with altered abundance of mitochondrial-dynamics, calcium-handling and PDH-regulatory proteins, with MFF the most strongly and reproducibly increased component and hence the natural candidate for validation; whether these abundance changes alter mitochondrial fission, fusion, calcium flux or pyruvate oxidation remains to be tested.

### 3.7. VEGF Does Not Alter the High-Glucose Signature: Direct Three-Group Contrasts

The Test+VEGF-vs.-Control comparison yielded 342 DAPs (185↑/157↓; 26 at q < 0.05) whose identity and direction closely mirrored the Test-vs.-Control set: 170 DAPs were shared between the two comparisons (Jaccard = 0.304; Figure 4b), and MFF remained increased (4.39-fold, *p* = 2.8 × 10^−4^). Because both comparisons share the Control group, we also analysed all nine samples in one three-group model with prespecified contrasts (Section 2.4; Table S7). The direct Test+VEGF-vs.-Test contrast identified 183 proteins at nominal *p* < 0.05 among 3289 tested, close to the ~164 expected by chance alone, and only nine at q < 0.05. MFF was unchanged by VEGF (0.91-fold, *p* = 0.71, q = 0.96), as were DRP1, FIS1, OPA1, MCU, MICU1, PDK3, ACADVL, NPC1, BNIP1 and the insulin-signalling proteins (all *p* > 0.05); GYS1 (0.13-fold, q = 0.006) was the only axis protein significantly altered by VEGF. Thus, at the proteome level VEGF neither normalised the high-glucose signature nor induced a broad distinct programme, mirroring our earlier functional finding that exogenous VEGF did not significantly restore insulin-stimulated glucose uptake in these cells [33]. Because no Control+VEGF arm was included, the design cannot test a glucose × VEGF interaction, and conclusions about VEGF are restricted to the high-glucose condition. This result also provides the rationale for the in vivo design below: because the in vitro VEGF effect was minimal, VEGF was not advanced to the animal model, and in vivo validation focused on the high-glucose-associated signature and its lead marker, MFF.

### 3.8. In Vivo Model: Streptozotocin Induces Hyperglycaemia in Pregnant Rats

**Figure 8:**
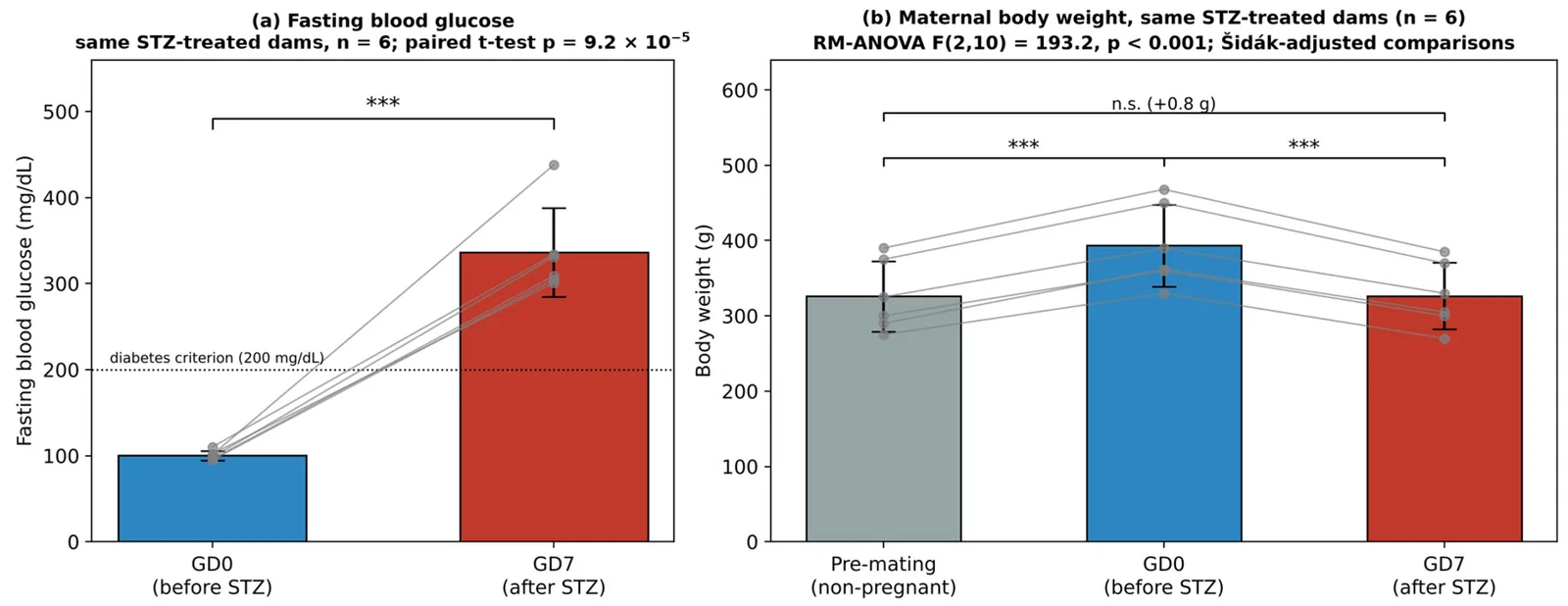
STZ-induced hyperglycaemia in pregnant Sprague-Dawley rats (the same six STZ-treated dams followed over time). (**a**) Fasting blood glucose at baseline (GD0, before STZ) and on GD7 (48 h after the last STZ dose); dotted line = 200 mg/dL diabetes criterion; *** paired *t*-test *p* < 0.001. (**b**) Body weight of the same dams before mating, at GD0 (before STZ) and on GD7; *** Šidák-adjusted *p* < 0.001; n.s., not significant (repeated-measures ANOVA F(2,10) = 193.2). Lines join values from the same dam; bars = mean ± SD. Vehicle-treated dams are not shown (see Section 3.8).

To provide an in vivo setting for candidate validation, hyperglycaemia was induced in pregnant Sprague-Dawley rats by streptozotocin (35 mg/kg body weight, i.p., GD1–GD5; ethics approval LPU/IAEC/2022/17), with vehicle-treated pregnant controls (Table 1). STZ induction was unambiguous: in the six STZ-treated dams, fasting blood glucose rose from 100.3 ± 5.5 mg/dL at baseline (GD0) to 336.3 ± 51.7 mg/dL on GD7 (paired mean difference +236.0 mg/dL, 95% CI 182.6 to 289.4; paired *t*-test *p* = 9.2 × 10^−5^), and every dam exceeded the 200 mg/dL criterion (Figure 8a). Body weight was followed in each dam from before mating to GD7 (Figure 8b; Table 8; Table S8) and differed across the three time points (repeated-measures ANOVA F(2,10) = 193.2, Greenhouse–Geisser-corrected *p* = 1.6 × 10^−7^). Every dam weighed more at GD0 than before mating (325.8 ± 47.1 to 393.3 ± 54.6 g; +67.5 g, Šidák-adjusted 95% CI 54.6 to 80.4; *p* < 0.001), and every dam then lost weight after STZ (to 326.7 ± 44.0 g on GD7; −66.7 g, Šidák-adjusted 95% CI −83.3 to −50.0; −17%; *p* < 0.001), so that by GD7 body weight had returned to the pre-mating value (difference +0.8 g, 95% CI −10.7 to 12.3; *p* = 0.99; every dam within 15 g of its pre-mating weight). STZ therefore reversed the entire weight gained between the pre-mating baseline and GD0 (mean 99% per dam), consistent with the catabolic state of insulin-deficient diabetic pregnancy. All glucose and weight comparisons are within-animal, each STZ-treated dam serving as its own control; the study records contain no glucose or weight values for the vehicle-treated dams, so the vehicle group serves as the comparator for serum MFF only (Section 3.9). Because STZ acts primarily by β-cell injury and insulin deficiency, this model reproduces maternal hyperglycaemia during pregnancy but not the insulin resistance that characterises human GDM [45,46,47]; we therefore refer to it as a model of hyperglycaemic pregnancy rather than of GDM.

### 3.9. ELISA Confirms Higher MFF in High-Glucose Cells and in Serum of Hyperglycaemic Pregnant Rats

**Figure 9:**
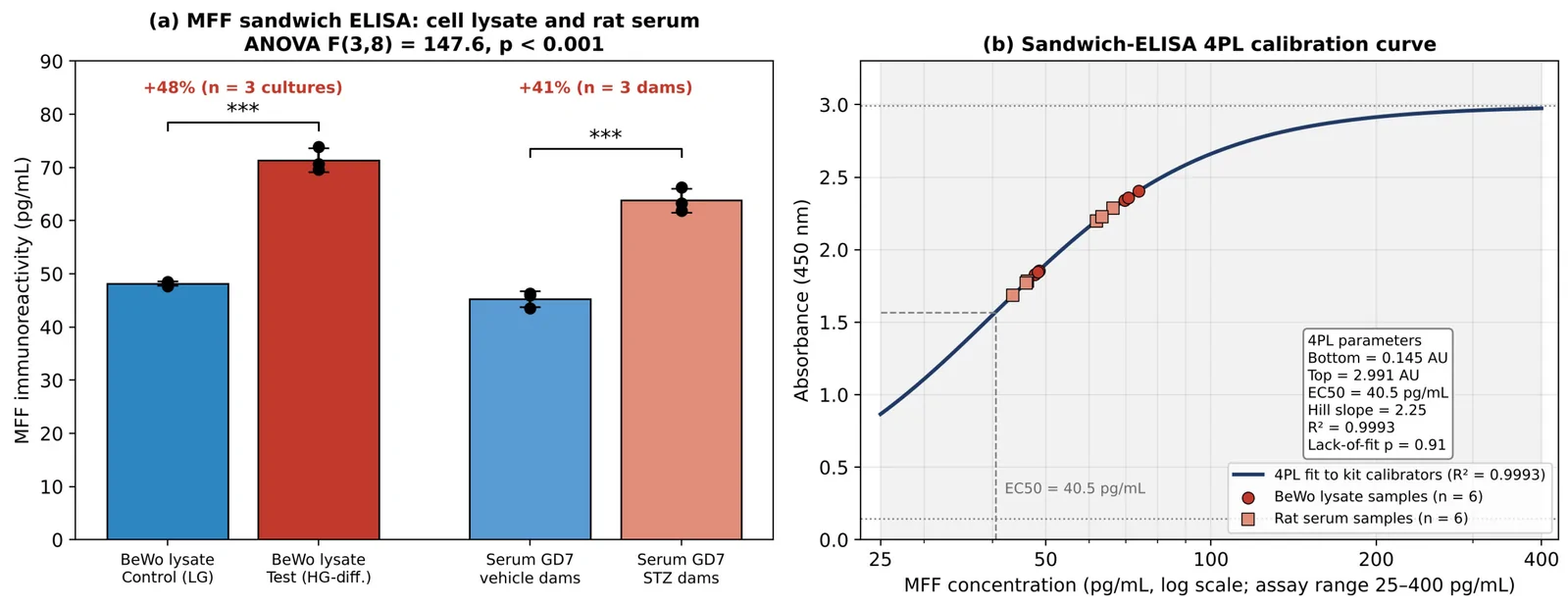
MFF sandwich-ELISA. (**a**) MFF in BeWo lysates (Control, undifferentiated in 5 mM glucose, vs. Test, syncytialised in 25 mM glucose; *n* = 3 independent cultures) and in GD7 serum of vehicle-treated vs. STZ-treated pregnant rats (*n* = 3 dams per group); bars = mean ± SD, points = individual samples; *** Šidák-adjusted *p* < 0.001 (one-way ANOVA F(3,8) = 147.6). Serum values are relative immunoreactivity against the human-calibrated kit. (**b**) 4PL calibration curve fitted to the kit calibrators over the assay range (25–400 pg/mL, shaded), with the fitted parameters (Bottom, Top, EC_50_, Hill slope, R^2^ = 0.9993, lack-of-fit *p* = 0.91) and the twelve interpolated samples (circles, BeWo lysate; squares, rat serum). All samples lie on the steep central segment of the curve. Note the similar ~40–48% increase in both matrices.

To confirm the lead proteomic candidate with an independent, antibody-based method, MFF was measured by sandwich-ELISA against a 4PL calibration curve fitted to the kit calibrators over the assay range of 25–400 pg/mL (R^2^ = 0.9993; lack-of-fit *p* = 0.91; Figure 9b). All twelve sample absorbances fell within the calibrated range, on the steep central segment of the curve where interpolation is most precise, and the rat-serum samples occupied the same region of the curve as the cell lysates. The four groups differed (one-way ANOVA F(3,8) = 147.6, *p* < 0.001, R^2^ = 0.9823), and Šidák-corrected comparisons confirmed both prespecified contrasts (Figure 9a; Table 8). In vitro, Test (25 mM) BeWo lysates contained more MFF than Control lysates (71.40 ± 2.24 vs. 48.21 ± 0.41 pg/mL; +48%; mean difference 23.2 pg/mL, Šidák-adjusted 95% CI 19.2 to 27.2, adjusted *p* < 0.001; Welch *t*-test *p* = 0.0024), corroborating the mass-spectrometry finding by an orthogonal method. In vivo, GD7 serum from STZ-treated hyperglycaemic dams showed higher MFF immunoreactivity than serum from vehicle-treated dams (63.81 ± 2.27 vs. 45.30 ± 1.53 pg/mL; +41%; mean difference 18.5 pg/mL, Šidák-adjusted 95% CI 14.5 to 22.5, adjusted *p* < 0.001; Welch *t*-test *p* = 6.2 × 10^−4^; *n* = 3 dams per group). The serum increase (+41%) was similar in magnitude to that in the cell model (+48%). The serum values are relative, kit-specific immunoreactivity (Section 2.6), the sample size is small, and serum was collected after hyperglycaemia had been established; the in vivo result therefore shows that MFF is associated with hyperglycaemic pregnancy in this model, not that it predicts GDM.

### 3.10. Integrated Summary of the Proteomic and ELISA Findings

**Figure 10:**
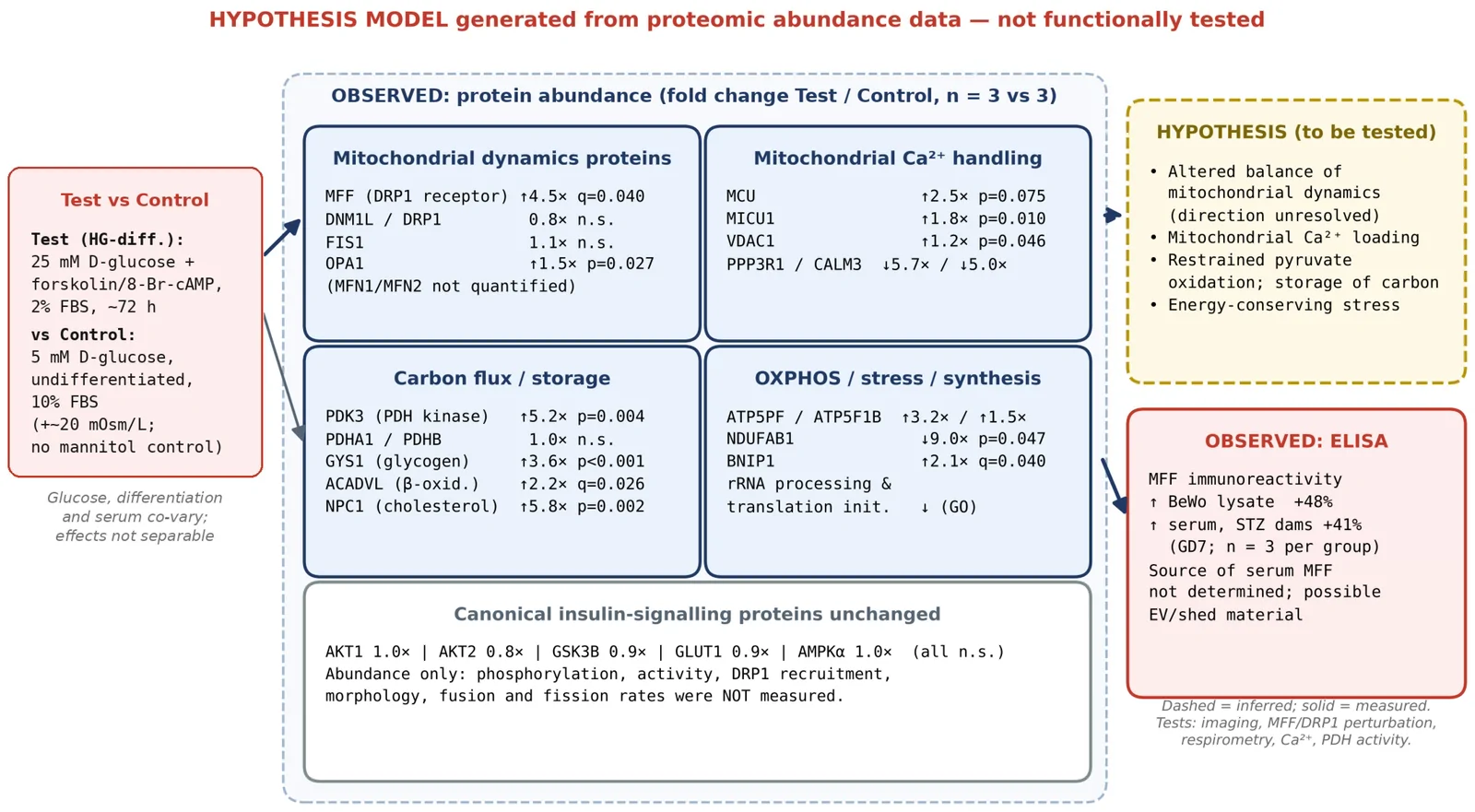
Hypothesis model generated from the proteomic findings (not functionally tested). The high-glucose differentiated state (25 mM glucose with forskolin/8-Br-cAMP and 2% FBS, ~72 h, versus undifferentiated cells in 5 mM glucose and 10% FBS; glucose, differentiation and serum vary together) is associated with increased abundance of the DRP1 receptor MFF with unchanged DRP1 and FIS1 and a modest rise in OPA1 (MFN1/MFN2 not quantified); increased mitochondrial calcium-uniporter components (MCU, MICU1, VDAC1) with decreased calcineurin-B and calmodulin-3; increased PDK3 with unchanged PDH subunits; increased GYS1, ACADVL and NPC1; and decreased protein-synthesis machinery, while canonical insulin-signalling proteins are unchanged in abundance. The hypothesised consequences (altered balance of mitochondrial dynamics of unresolved direction, mitochondrial calcium loading, restrained pyruvate oxidation) are shown with dashed outlines and require testing by mitochondrial imaging, MFF/DRP1 perturbation, respirometry, and calcium and PDH-activity measurements. MFF immunoreactivity was confirmed by ELISA in cell lysate (+48%) and in GD7 serum of STZ-treated dams (+41%); the tissue source of serum MFF was not determined (possible routes, [29,30,31]). Fold changes are Test/Control from Table 7. ↑, increased; ↓, decreased abundance; n.s., not significant.

Figure 10 brings the proteomic and ELISA results together. It separates what was measured, namely protein abundance in the cell model and MFF immunoreactivity in cell lysates and rat serum, from what can only be inferred at present, such as the direction of mitochondrial dynamics or the tissue source of serum MFF. The figure should be read as a hypothesis generated by the data, not as a demonstrated mechanism; the inferred elements are discussed in Section 4.3.

## 4. Discussion

### 4.1. Main Findings

**Figure 11:**
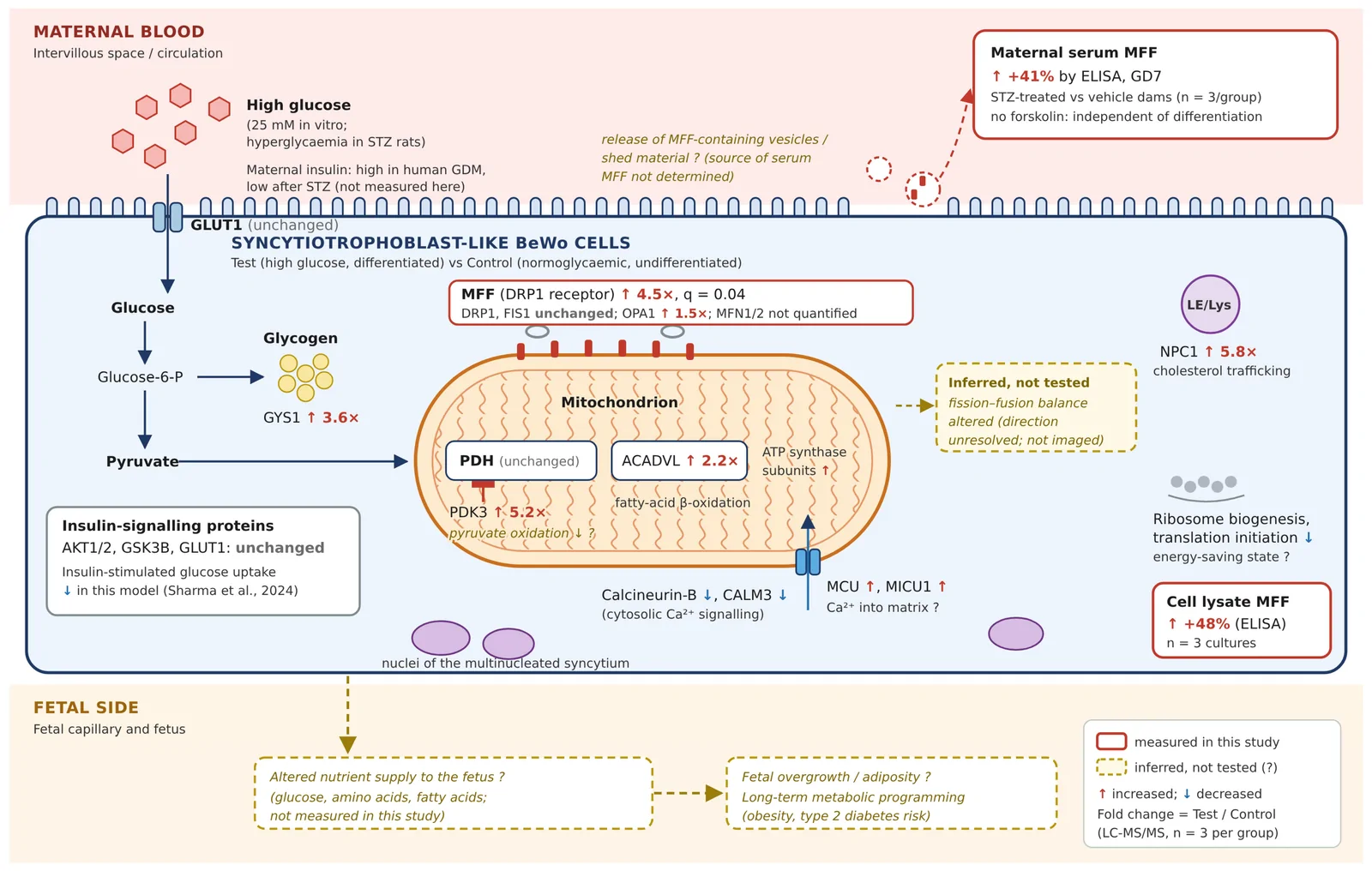
Summary of the main findings from a metabolic and cellular perspective. In the high-glucose differentiated BeWo model (Test vs. Control), MFF increased while DRP1 and FIS1 did not change; the mitochondrial calcium-uniporter components MCU and MICU1 increased and calcineurin-B (PPP3R1) and calmodulin-3 (CALM3) decreased; PDK3 increased with unchanged PDH subunits; GYS1, ACADVL and NPC1 increased; and ribosome-biogenesis and translation-initiation proteins decreased. Canonical insulin-signalling proteins (AKT1/2, GSK3B, GLUT1) were unchanged in abundance, although insulin-stimulated glucose uptake is reduced in this model [33]. MFF was higher by ELISA in cell lysates and in serum of STZ-treated hyperglycaemic pregnant rats. Solid outlines and arrows mark measured changes; dashed outlines and arrows mark inferred, untested consequences: altered mitochondrial dynamics, restrained pyruvate oxidation, release of MFF-containing material into maternal blood, altered nutrient supply to the fetus and possible effects on fetal growth and later metabolic health.

We set out to describe the proteome of an established insulin-resistant trophoblast model and to follow one candidate from discovery to a serum measurement in vivo. Because glucose, syncytialisation and serum concentration changed together in this model [33], the in vitro results describe the high-glucose differentiated state rather than an isolated effect of glucose (Section 4.7, Limitation i). The design still suits biomarker discovery. The syncytiotrophoblast is the layer that sheds placental material into maternal blood [29,30,31], so the Test condition stands in for the hyperglycaemic syncytium from which a circulating marker would come, and the rat serum data ask whether the candidate follows hyperglycaemia without any in vitro differentiation. Four results stand out. First, the proteome changed in a coordinated way along mitochondrial and metabolic lines, while the overall protein complement stayed largely the same. Second, among the mitochondrial-dynamics proteins the change was selective for the DRP1 receptor MFF; DRP1 and FIS1 did not change. Third, VEGF left this pattern intact, in keeping with its lack of functional effect in the same model [33]. Fourth, MFF was higher by ELISA in high-glucose cell lysates and in serum of hyperglycaemic pregnant rats. Figure 11 summarises these findings from a metabolic and cellular point of view.

### 4.2. Proteomics and Gestational Diabetes

Proteomic studies of GDM have so far examined term placental tissue, circulating exosomes and maternal plasma [7,8,34,35,36,37,38]. A meta-analysis of 24 such studies found 22 candidate proteins that replicated across platforms, mostly in blood and mostly linked to the complement and coagulation cascades [39], and a recent systematic review of placental omics in maternal diabetes found only eight proteins that changed consistently in two or more studies, with immune, vascular and developmental pathways the most affected [49]. Small cohorts, different platforms and limited validation are common to this literature. Our study differs in two ways. It follows a candidate from unbiased, FDR-controlled discovery in one defined cell system, through an independent antibody-based assay, to a serum measurement in vivo; and the candidate it found, MFF, has independently been reported to be more abundant in placentae from women with GDM [28]. If confirmed in prospective human samples, MFF could add a mitochondrial, and therefore potentially mechanistic, dimension to early-pregnancy candidates such as apolipoprotein CIII, SHBG and multi-protein plasma panels [7,8,38]. For now, the in vivo evidence rests on three dams per group in an STZ model at a single time point after hyperglycaemia had developed.

### 4.3. Mitochondrial Dynamics, Energy Metabolism and Trophoblast Development

MFF is a plausible candidate for the reasons outlined in the Introduction: it is the main receptor that recruits DRP1 to mitochondria [23], and AMPK phosphorylates it under energy stress, linking fuel supply to fission [24]. A selective 4.5-fold rise in MFF with unchanged DRP1 and FIS1 is therefore compatible with a greater capacity to recruit DRP1. These are abundance data, however. They do not show that fission is increased, that MFF is rate-limiting or that DRP1 is recruited, and the modest rise in OPA1 means that the net direction of mitochondrial dynamics cannot be inferred. Human data counsel the same caution. Term GDM placentae show dynamics tilted toward fusion, perhaps as an adaptation to maternal and fetal hyperinsulinaemia [27], and mitochondrial dysfunction in both cytotrophoblast and syncytiotrophoblast [26]. Our model applies about 72 h of high glucose together with differentiation, without the insulin exposure, duration and cellular mix of the term placenta, and syncytialisation alone changes trophoblast mitochondrial mass and respiration [57,58].

Several findings indicate that glucose contributes to the MFF signal. Easton et al. compared 25 mM with control glucose for 72 h in BeWo cells kept at the same differentiation state and found more fission-associated markers, larger glycogen and triglyceride stores and unchanged respiration [50]; this matches our increases in MFF, GYS1, ACADVL and NPC1. Wei et al. found that high glucose raised MFF and DRP1 in HTR8/SVneo trophoblasts, which had not been treated with forskolin, and that MFF was more abundant in placentae from women with GDM than from normoglycaemic women (*n* = 20 per group); in their cells, MFF-associated fission went together with impaired insulin signalling and lower glucose uptake [28], much like the insulin-resistant phenotype of our model [33]. In addition, forskolin and 8-Br-cAMP act through cAMP–PKA signalling, which phosphorylates DRP1 at Ser637, inhibits its GTPase activity and elongates mitochondria [59,60]. The differentiation stimulus would therefore be expected to work against a fission-associated signature rather than create one. Our unbiased screen thus arrived at the same protein that a candidate-driven study found raised in human GDM placentae.

The other changes describe how the cells handle fuel. More MCU and MICU1, together with less calcineurin-B and calmodulin-3, would be expected to shift calcium toward the mitochondrial matrix, which can itself promote DRP1-dependent fission and superoxide production [54]. A rise in PDK3 with unchanged PDH catalytic subunits points to restrained pyruvate oxidation [55], and more GYS1 suggests that glucose that is not oxidised is diverted into glycogen, a recognised feature of the diabetic placenta [11,50]. Higher ACADVL and NPC1 point to greater use of fatty acids and altered cholesterol handling, and the loss of ribosome-biogenesis and translation-initiation proteins fits an energy-saving state in which protein synthesis is cut back. Put simply, these cells appear to take up glucose but to store rather than burn it. This could explain why insulin-stimulated glucose uptake was impaired in the same model [33] even though AKT1/2, GSK3B and GLUT1 were unchanged. The same pathways matter for development. Trophoblast differentiation depends on mitochondrial function [57], placental mitochondrial respiration changes across gestation and in complicated pregnancies [61,62], and the hormone synthesis and active transport of the syncytiotrophoblast both rely on mitochondria [19]. A recent systematic review of placental omics in maternal diabetes likewise found developmental pathways among those most consistently disturbed [49]. Lasting changes in mitochondrial dynamics and fuel use could therefore affect how the placenta grows and works, and not only serve as a marker. Each of these inferences still needs functional testing (Limitation v). Mitochondrial dysfunction is also shared with other metabolic disorders, including metabolic dysfunction-associated steatotic liver disease, where it has been targeted nutritionally [63], although the relevance to GDM and to MFF is indirect.

Mitochondrial clustering, mitochondrial fusion and trophoblast syncytialisation must be clearly distinguished. Mitochondrial fusion is the merging of outer and inner membranes with mixing of matrix content, which requires the mitofusins MFN1/MFN2 on the outer membrane and OPA1 on the inner membrane [64,65]; mitochondria can also become tethered, closely apposed or aggregated (‘clumped’) without fusing, and clumping can accompany impaired rather than enhanced fusion [66]. Altered abundance of dynamics proteins could in principle be associated with such clustering. However, MFN1/MFN2 were not quantified here, and neither mitochondrial morphology nor content mixing was assessed; any link between the observed protein changes and mitochondrial clustering or fusion therefore remains hypothetical. Confocal imaging of mitochondrial networks, with quantification of clustering, branch length and fragmentation, and a content-mixing assay (for example, photoactivatable matrix-targeted GFP) would be needed to distinguish these states. Finally, the multinucleated sheets and cell aggregates in Figure 1 reflect cell-level syncytialisation, i.e., fusion of trophoblast plasma membranes, and do not report mitochondrial clustering or fusion.

### 4.4. Placental Nutrient Handling, Maternal Obesity and Metabolic Programming

GDM matters beyond pregnancy because of its long-term effects on mother and child. In the HAPO Follow-up Study, women who had GDM had 3.4-fold higher odds of type 2 diabetes or prediabetes 10–14 years later, and their children were more likely to be obese, even after adjustment for maternal body mass index [67]. Maternal obesity has similar consequences, raising the offspring’s risk of obesity, type 2 diabetes and cardiovascular disease, partly through epigenetic mechanisms [68], and obesity and GDM often occur together [5,9]. The placenta sits at the centre of this metabolic programming. It senses the maternal nutrient supply and adjusts nutrient transport and growth signals to the fetus, so changes in its function can alter fetal growth and set the course of later metabolic health [69]. In GDM, placental transport of glucose, amino acids and fatty acids is altered and has been linked to fetal overgrowth and fat accretion [70], and the placenta stays responsive to insulin, so that maternal hyperinsulinaemia can increase its amino-acid transport [71]. Our data add a mitochondrial side to this picture. The syncytiotrophoblast pays for active nutrient transport and hormone synthesis with mitochondrial ATP [19]. A trophoblast that restrains pyruvate oxidation, stores glycogen and remodels its mitochondria, as suggested here, may handle and pass on nutrients differently, with possible effects on fetal growth and later disease risk (Figure 11). This link is speculative, because we did not measure nutrient transport, fetal growth or offspring outcomes. It does, however, place MFF and the associated proteins within the wider question of how GDM programmes long-term health, and it points to outcomes worth measuring in future studies.

### 4.5. Serum MFF as a Candidate Biomarker

Why should a change in a trophoblast outer-mitochondrial-membrane protein be detectable in maternal serum? There are three reasons to expect it. First, the syncytiotrophoblast is a secretory epithelium bathed directly in maternal blood that continuously sheds syncytial knots, microvesicles and exosomes carrying cytoplasmic and organellar proteins; placenta-derived vesicles are readily detected in maternal plasma from early gestation, and both their concentration and their protein cargo are altered in GDM [29,30,31]. Mitochondrial proteins, including outer-membrane components, are recognised constituents of this shed material, and a mitochondrial-stress phenotype such as that suggested here (MFF, BNIP1 and calcium-uniporter increases) is a condition under which mitochondrial content may enter extracellular vesicles. Second, hyperglycaemia is systemic: STZ-induced maternal hyperglycaemia and insulin deficiency expose liver, skeletal muscle and adipose tissue, which is itself metabolically and inflammatorily activated in GDM [72], to the same glucose excess that promotes mitochondrial fission in these tissues [17,18], so circulating MFF in the whole-animal model plausibly integrates a placental with a maternal-tissue contribution. Third, the effect size in serum (+41%) was similar to that in the high-glucose differentiated trophoblast cultures (+48%), the pattern expected if serum MFF reports a related glucose-associated cellular response. Because the rats received no forskolin or 8-Br-cAMP, the higher serum MFF in STZ-treated dams is evidence, independent of in vitro differentiation, that MFF is associated with hyperglycaemia. We did not measure serum insulin, so we cannot say how much of the serum signal reflects insulin deficiency rather than glucose itself. This matters because insulin is usually high, not low, in human GDM, and maternal insulin acts directly on the placenta [71]; the STZ result therefore needs confirmation in an insulin-resistant model and in women (Limitation vii). We emphasise that the present design cannot apportion circulating MFF between placental and maternal sources, that a single GD7 time point after the onset of hyperglycaemia was sampled, and that serum values are relative immunoreactivity with a human-calibrated kit; these points are addressed in the Limitations below. For biomarker purposes, what matters at this stage is that a candidate found by unbiased trophoblast proteomics behaves in the same way in an accessible maternal biofluid under gestational hyperglycaemia, which is what is needed before testing in human cohorts.

### 4.6. Effect of VEGF

VEGF signalling is altered in GDM, with increased pro-migratory VEGFR2 activation and reduced VEGFR1 expression in the placenta [73], and growth factors regulate trophoblast function through receptor-specific pathways [74]; we therefore asked whether VEGF modifies the high-glucose phenotype. VEGF was evaluated in vitro (Test+VEGF group) and produced no statistically significant effect on insulin-stimulated glucose uptake [33] and minimal effect on the trophoblast proteome (direct Test+VEGF-vs.-Test contrast: 183 nominal DAPs among 3289 proteins, close to chance expectation; MFF 0.91-fold, *p* = 0.71). Because VEGF had so little effect in vitro, it was not carried forward into the STZ model. The VEGF-treated cultures were still useful, serving as a second set of high-glucose cultures for the mitochondrial–metabolic proteins (Table 7).

### 4.7. Limitations and Future Directions

Several limitations should be acknowledged. (i) Design: in this established model [33], high glucose was applied together with forskolin/8-Br-cAMP-induced syncytialisation and a reduction of serum from 10% to 2%, whereas Control cells remained undifferentiated in LG medium. The Test-versus-Control differences in the proteome and in lysate MFF therefore reflect the combined high-glucose differentiated condition and cannot be attributed to glucose alone; part of the mitochondrial signature may arise from syncytialisation itself, which remodels trophoblast mitochondrial mass and respiration [57,58]. Several observations nevertheless support a glucose-associated component of the MFF signal: high glucose increases MFF in non-syncytialised HTR8/SVneo trophoblasts, and MFF is elevated in human GDM placentae [28]; fission-associated markers increase with 25 mM glucose at a constant differentiation state in BeWo cells [50]; serum MFF was higher in STZ-treated hyperglycaemic rats, which received no forskolin; the cAMP–PKA pathway engaged by the differentiation stimulus inhibits, rather than promotes, DRP1-dependent fission [59,60]; and no serum-deprivation response was detectable in the proteome (Section 3.3). Even so, differentiated normoglycaemic and undifferentiated high-glucose controls with serum-matched media are required to separate the three factors. (ii) A single choriocarcinoma-derived cell line was used; although syncytialised BeWo cells are a widely used tractable model of the syncytiotrophoblast [32] and the present model was functionally characterised [33], confirmation in primary trophoblasts or trophoblast organoids is required, and STR re-authentication and mycoplasma testing were not documented for the cultures used here. (iii) The design is compact (*n* = 3 independent cultures per group for proteomics and ELISA; *n* = 6 STZ-treated dams for glucose and body weight; *n* = 3 dams per group for serum ELISA), which limits power and precision; we therefore rely on FDR control, effect sizes with confidence intervals, orthogonal confirmation and concordance checks rather than on nominal *p*-values alone. (iv) Only 25 mM versus 5 mM glucose at a single ~72 h time point was tested, without an iso-osmolar mannitol control, a glucose dose–response or a time course. The 20 mM glucose increment adds ~20 mOsm/L (≈7%); AQP1, a hypertonicity-responsive protein, was modestly increased (2.2-fold, nominal *p* = 0.003, q = 0.13), whereas aldose reductase (AKR1B1) and the stress chaperones HSPA5, HSPA8, HSPH1, DNAJB1 and HMOX1 were unchanged (Table S9). A classical hyperosmotic-stress response is therefore not evident, but an osmotic contribution cannot be excluded, and the signature should be read as a response to the tested high-glucose differentiated condition. Viability was > 96% in our previous characterisation of the model [33] but was not measured separately for each condition in the proteomic cultures, and the data are normalised to total protein rather than cell number. (v) The mechanistic interpretation (Figure 10) rests on protein abundance and co-abundance; abundance does not report phosphorylation, activity, DRP1 recruitment, mitochondrial morphology, membrane potential, respiration, reactive oxygen species, calcium flux or PDH activity, none of which was measured, and no MFF/DRP1 loss-of-function or pharmacological perturbation was performed. Mitochondrial imaging, a content-mixing fusion assay, MFF knock-down or DRP1 inhibition, respirometry, calcium imaging and PDH-activity assays are the logical next experiments. (vi) The VEGF arm shares the Control group and no Control+VEGF arm was included, so a glucose × VEGF interaction cannot be tested. (vii) STZ produces hyperglycaemia mainly through β-cell injury and insulin deficiency, with systemic metabolic consequences that differ from the insulin-resistant state of human GDM [45,46,47]; our in vivo data therefore model hyperglycaemic pregnancy, not GDM. Serum insulin, C-peptide and HOMA-IR were not measured, because the in vivo arm was designed to test the hyperglycaemia-associated serum signal rather than to characterise the metabolic state of the dams; the degree of insulin deficiency was therefore inferred from the known action of STZ and from the weight loss after STZ, not measured directly, and future studies should include these measurements. (viii) Serum MFF was measured at a single time point (GD7) after hyperglycaemia had been established, in three dams per group, with a human-calibrated kit whose rat-matrix performance (spike-recovery, dilution linearity) was not validated; the result shows association, not predictive or diagnostic performance [48], and the tissue source of circulating MFF (placenta versus maternal tissues) cannot be assigned. Placental-tissue MFF, placenta-derived extracellular-vesicle fractionation, a gestational time course beginning before hyperglycaemia and an insulin-resistant model are needed. (ix) ELISA was the sole orthogonal assay and only the lead candidate was validated; PDK3, MICU1 and BNIP1 are second-tier candidates. Finally, any clinical use requires an adequately powered prospective human cohort, with first-trimester sampling before diagnosis and formal diagnostic-performance metrics (sensitivity, specificity, ROC-AUC); until then, MFF should be regarded as a candidate biomarker associated with hyperglycaemic pregnancy.

## 5. Conclusions

Label-free proteomics of an established insulin-resistant BeWo trophoblast model, in which cells syncytialised in high-glucose medium are compared with undifferentiated normoglycaemic cells, shows altered abundance of mitochondrial-dynamics, calcium-handling and carbon-flux proteins and reduced protein-synthesis machinery, without changes in the abundance of canonical insulin-signalling proteins. Because glucose, differentiation and serum varied together, these changes describe the high-glucose differentiated state rather than an isolated effect of glucose. Within this signature, mitochondrial fission factor (MFF) is the most strongly and reproducibly increased component: it survives FDR correction, is selective relative to DRP1 and FIS1, behaves concordantly in a second set of high-glucose cultures, is unaffected by VEGF, and is confirmed by ELISA in cell lysates and, independently of in vitro differentiation, in serum of STZ-treated hyperglycaemic pregnant rats. This unbiased finding converges with the recently reported increase of MFF in human GDM placentae and in glucose-exposed trophoblasts that were not differentiated [28]. These abundance and association data generate, rather than establish, the hypothesis that trophoblast mitochondrial dynamics are altered under high glucose, and they identify MFF as a candidate serum biomarker associated with hyperglycaemic pregnancy. Studies that separate glucose from differentiation, functional studies of mitochondrial dynamics, and prospective evaluation in human cohorts with sampling before the onset of hyperglycaemia are required before any predictive or diagnostic role can be claimed.

## Figures and Tables

**Table 1:** In vivo experimental design (STZ-induced hyperglycaemic pregnant-rat model).

Group (Pregnant SD Rat)	Induction	Route/Dose
Vehicle control (6 pregnant ♀; 2 ♂ for mating)	Vehicle only	0.1 M citrate buffer, i.p., once daily GD1–GD5; serum on GD7 (ELISA, *n* = 3)
STZ, hyperglycaemic (6 pregnant ♀; 2 ♂ for mating)	Streptozotocin (STZ)	35 mg/kg b.w., i.p., once daily GD1–GD5; glucose and body weight at GD0 and GD7 (*n* = 6); serum on GD7 (ELISA, *n* = 3)

**Table 2:** Proteome identification, quality-control and differential-abundance summary.

Metric	Value
MS runs (Orbitrap Exploris, 60 min)	9 (3 groups × 3 biological replicates)
Protein groups identified (≤1% FDR)	3528
Peptide groups identified	22,431
Core proteins shared by all groups	3357 (95.2%)
Proteins unique to Control/Test/Test+VEGF	31/15/31
Median within-group correlation (DAP set, z-scored)	0.63
Median between-group correlation (DAP set, z-scored)	−0.73
PCA variance explained, DAP set (PC1/PC2)	74.3%/8.7%
PCA PC1, all 3271 proteins (unsupervised)	32.8%; complete Test/Control separation
DAPs Test vs. Control (*p* < 0.05)	388 (208 ↑/180 ↓)
DAPs Test vs. Control (BH q < 0.05/q < 0.10)	24/47
DAPs Test+VEGF vs. Control (*p* < 0.05)	342 (185 ↑/157 ↓)
Shared DAPs between both comparisons (Jaccard)	170 (0.304)
Three-group model: Test vs. Control (*p* < 0.05/q < 0.05)	451/28
Three-group model: Test+VEGF vs. Test (*p* < 0.05/q < 0.05)	183/9 (≈164 expected by chance)

↑/↓, number of proteins with increased/decreased abundance relative to Control (*p* < 0.05); DAP, differentially abundant protein; BH, Benjamini–Hochberg.

**Table 3:** Proteins significant after Benjamini–Hochberg FDR correction (q < 0.05; Test vs. Control).

Symbol	FC	Dir	*p*	q	Symbol	FC	Dir	*p*	q
RND3	34.17	↑	3.3 × 10^−6^	0.006	GNE	2.74	↑	1.4 × 10^−4^	0.034
VTI1B	12.22	↑	3.5 × 10^−6^	0.006	CDK6	0.5	↓	1.5 × 10^−4^	0.035
NCK1	0.34	↓	9.5 × 10^−6^	0.010	SMARCD1	2.73	↑	1.8 × 10^−4^	0.039
PTPA	6.55	↑	1.2 × 10^−5^	0.010	SNAP23	2.04	↑	2.3 × 10^−4^	0.040
UTP20	0.14	↓	1.9 × 10^−5^	0.011	TRIO	1.91	↑	2.4 × 10^−4^	0.040
ITPR1	2.63	↑	2.0 × 10^−5^	0.011	BNIP1	2.14	↑	2.5 × 10^−4^	0.040
SNTB2	2.65	↑	4.4 × 10^−5^	0.021	ACOT7	0.53	↓	2.6 × 10^−4^	0.040
RBMS2	0.29	↓	6.5 × 10^−5^	0.024	QKI	2.04	↑	2.6 × 10^−4^	0.040
RAB11FIP5	0.34	↓	6.6 × 10^−5^	0.024	ARHGEF17	3.92	↑	2.7 × 10^−4^	0.040
CTBP2	0.2	↓	8.1 × 10^−5^	0.025	MFF	4.52	↑	2.8 × 10^−4^	0.040
MAN1A2	3.54	↑	8.5 × 10^−5^	0.025	FAM114A2	4.35	↑	2.9 × 10^−4^	0.040
ACADVL	2.2	↑	9.6 × 10^−5^	0.026	QDPR	0.37	↓	3.0 × 10^−4^	0.040

FC, linear fold change (2^log2FC^, Test/Control); Dir, direction (↑, increased; ↓, decreased in Test vs. Control); *p*, limma moderated *t*-test *p*-value; q, BH-adjusted *p*-value. Twenty-four proteins in total; MFF is highlighted in the main text.

**Table 4:** Representative differentially abundant proteins (Test vs. Control).

Symbol	Protein	Fold Change	Dir.	*p*-Value
RND3	Rho-related GTP-binding protein RhoE	34.17	↑	3.3 × 10^−6^
MFF	Mitochondrial fission factor	4.52	↑	2.8 × 10^−4^
PDK3	[Pyruvate dehydrogenase (acetyl-transferring)] kinase isozyme 3, mitochondrial	5.24	↑	3.9 × 10^−3^
NPC1	NPC intracellular cholesterol transporter 1	5.79	↑	1.8 × 10^−3^
GYS1	Glycogen [starch] synthase, muscle	3.58	↑	5.7 × 10^−4^
MCCC1	Methylcrotonoyl-CoA carboxylase subunit alpha, mitochondrial	3.51	↑	1.1 × 10^−2^
ATP5PF	ATP synthase-coupling factor 6, mitochondrial	3.20	↑	7.7 × 10^−3^
SLC25A11	Mitochondrial 2-oxoglutarate/malate carrier protein	1.68	↑	3.8 × 10^−3^
H3-3A/H3-3B	Histone H3.3	20.51	↑	1.2 × 10^−3^
GOLPH3	Golgi phosphoprotein 3	4.63	↑	4.0 × 10^−4^
NDUFAB1	Acyl carrier protein, mitochondrial	0.11	↓	4.7 × 10^−2^
PPP3R1	Calcineurin subunit B type 1	0.18	↓	6.2 × 10^−4^
CALM3	Calmodulin-3	0.20	↓	1.8 × 10^−2^
AFP	Alpha-fetoprotein	0.30	↓	3.8 × 10^−3^
ALDH7A1	Alpha-aminoadipic semialdehyde dehydrogenase	0.19	↓	8.8 × 10^−3^
CTBP2	C-terminal-binding protein 2	0.20	↓	8.1 × 10^−5^
SSBP1	Single-stranded DNA-binding protein, mitochondrial	0.22	↓	4.8 × 10^−4^
UTP20	Small subunit processome component 20 homolog	0.14	↓	1.9 × 10^−5^
BAG1	BAG family molecular chaperone regulator 1	0.18	↓	1.6 × 10^−3^
YBX3	Y-box-binding protein 3	0.24	↓	2.6 × 10^−2^

↑/↓, direction of change under high glucose (25 mM). Fold change is the linear fold change (2^log2FC^); *p*-value from the limma moderated *t*-test (*n* = 3 per group).

**Table 5:** Selected KEGG pathway enrichment (Test vs. Control).

Dir.	KEGG Pathway	*n*	*p*-Value	Fold enr.	BH-FDR
*Up*	Insulin resistance (hsa04931)	5	0.068	3.2	n.s.
*Up*	Diabetic cardiomyopathy (hsa05415)	8	0.026	2.7	n.s.
*Up*	Cholesterol metabolism (hsa04979)	4	0.035	5.5	n.s.
*Up*	Glycerolipid metabolism (hsa00561)	4	0.064	4.3	n.s.
*Up*	Val/Leu/Ile degradation (hsa00280)	4	0.030	5.8	n.s.
*Up*	Spinocerebellar ataxia (hsa05017)	9	9.7 × 10^−4^	4.4	0.22
*Down*	RNA degradation (hsa03018)	8	2.8 × 10^−5^	8.9	0.006 *
*Down*	Aminoacyl-tRNA biosynthesis (hsa00970)	4	0.039	5.3	n.s.
*Down*	Biosynthesis of amino acids (hsa01230)	4	0.054	4.6	n.s.
*Down*	Ubiquitin-mediated proteolysis (hsa04120)	5	0.079	3.1	n.s.

* Survives Benjamini–Hochberg FDR < 0.05; n.s., not significant after correction. Uncorrected pathways are hypothesis-generating and interpreted alongside the FDR-controlled differential-abundance results.

**Table 6:** Selected Gene Ontology enrichment (Test vs. Control).

Ont.	Enriched Term	Dir.	*p*-Value	Fold enr.	BH-FDR
*CC*	Nuclear envelope	Up	5.3 × 10^−12^	8.9	2.3 × 10^−9^
*CC*	Mitochondrion	Up	2.7 × 10^−9^	2.8	3.9 × 10^−7^
*CC*	Mitochondrial outer membrane	Up	6.3 × 10^−8^	6.7	5.0 × 10^−6^
*CC*	Endoplasmic reticulum	Up	3.0 × 10^−8^	2.9	3.0 × 10^−6^
*BP*	Positive regulation of cell growth	Up	3.7 × 10^−5^	8.7	0.035
*BP*	Calcium import into mitochondrion	Up	4.6 × 10^−4^	25.5	0.11
*BP*	Mitochondrial fusion	Up	1.3 × 10^−3^	18.2	0.27
*MF*	Cholesterol binding	Up	2.9 × 10^−3^	8.4	0.40
*MF*	RNA binding	Down	1.3 × 10^−11^	3.2	4.9 × 10^−9^
*BP*	rRNA processing	Down	1.8 × 10^−5^	7.9	0.018
*BP*	Translational initiation	Down	1.4 × 10^−3^	10.2	0.37

CC, Cellular Component; BP, Biological Process; MF, Molecular Function. Representative non-redundant terms; full lists in Supplementary Data. BH-FDR, Benjamini–Hochberg-adjusted *p*-value. ‘Mitochondrial fusion’ comprises OPA1, MFF, AFG3L2 and CHCHD3, and ‘calcium import into the mitochondrion’ comprises ITPR1, AFG3L2, VDAC1 and MICU1; neither survives FDR correction, and GO annotation does not demonstrate a change in the corresponding process.

**Table 7:** Abundance of mitochondrial-dynamics, calcium-handling, carbon-flux and insulin-signalling proteins in the existing quantitative dataset.

Functional Node	Symbol	Protein	FC Test/Ctrl	*p* (Test/Ctrl)	FC Test+VEGF/Ctrl	*p* (Test+VEGF/Ctrl)
Fission–fusion	MFF	Mitochondrial fission factor (DRP1 receptor)	4.52 ↑	2.8 × 10^−4^ (q = 0.040)	4.39 ↑	2.8 × 10^−4^
	DNM1L	Dynamin-1-like protein (DRP1)	0.84	0.13 n.s.	0.80	0.10 n.s.
	FIS1	Mitochondrial fission 1 protein	1.12	0.31 n.s.	1.03	0.85 n.s.
	OPA1	Dynamin-like 120 kDa protein (fusion)	1.54 ↑	0.027	1.52	0.062
Mitochondrial Ca^2+^	MCU	Calcium uniporter protein	2.54	0.075	2.66 ↑	0.0084
	MICU1	Calcium uptake protein 1	1.80 ↑	0.0098	2.12 ↑	0.0073
	VDAC1	Voltage-dependent anion channel 1	1.22 ↑	0.046	1.22	0.10 n.s.
	PPP3R1	Calcineurin subunit B type 1	0.18 ↓	6.2 × 10^−4^	0.28 ↓	0.0071
	CALM3	Calmodulin-3	0.20 ↓	0.018	0.18 ↓	0.0079
PDH node/carbon flux	PDK3	Pyruvate dehydrogenase kinase 3	5.24 ↑	0.0039	4.09 ↑	0.0090
	PDHA1	PDH E1 component subunit α	0.98	0.79 n.s.	1.01	0.93 n.s.
	PDHB	PDH E1 component subunit β	1.16	0.12 n.s.	1.19	0.13 n.s.
	PDP1	PDH phosphatase catalytic subunit 1	0.72	0.34 n.s.	0.95	0.68 n.s.
	GYS1	Glycogen synthase, muscle	3.58 ↑	5.7 × 10^−4^	0.48 ↓	0.0022
	ACADVL	Very-long-chain acyl-CoA dehydrogenase	2.20 ↑	9.6 × 10^−5^ (q = 0.026)	2.55 ↑	4.7 × 10^−4^
	NPC1	NPC intracellular cholesterol transporter 1	5.79 ↑	0.0018	8.55 ↑	6.0 × 10^−4^
OXPHOS/stress	ATP5PF	ATP synthase coupling factor 6	3.20 ↑	0.0077	1.46	0.44 n.s.
	NDUFAB1	Acyl carrier protein, mitochondrial	0.11 ↓	0.047	0.10 ↓	0.038
	SOD2	Superoxide dismutase [Mn], mitochondrial	0.82	0.10 n.s.	0.57 ↓	0.0050
	BNIP1	Vesicle transport protein SEC20 (BNIP1)	2.14 ↑	2.5 × 10^−4^ (q = 0.040)	2.07 ↑	0.0019
Insulin signalling	AKT1	RAC-alpha serine/threonine-protein kinase	0.99	0.93 n.s.	1.16	0.35 n.s.
	AKT2	RAC-beta serine/threonine-protein kinase	0.77	0.18 n.s.	0.76	0.23 n.s.
	GSK3B	Glycogen synthase kinase-3 β	0.91	0.40 n.s.	0.92	0.79 n.s.
	SLC2A1	Glucose transporter GLUT1	0.93	0.45 n.s.	1.06	0.63 n.s.
	PRKAA1	AMPK catalytic subunit α-1	1.03	0.77 n.s.	0.94	0.71 n.s.

FC, linear fold change (2^log2FC^) from the limma moderated *t*-test (*n* = 3 per group); ↑/↓ mark nominal *p* < 0.05; n.s., not significant; q, Benjamini–Hochberg-adjusted *p* where the protein is among the 24 FDR-significant proteins. The Test+VEGF columns are a second set of three high-glucose cultures compared with the same Control group (MFF quantified in two of the three Test+VEGF cultures). The direct Test+VEGF-vs.-Test contrast from the three-group model is given in Table S7. MFN1/MFN2 were not quantified in this dataset. All values are abundance measurements from the existing dataset; no activity, phosphorylation or morphology was measured, and no additional experiments were performed.

**Table 8:** In vivo model and MFF ELISA results (all measured in this study), with effect sizes.

Parameter (Unit; *n*)	Group 1 (Mean ± SD)	Group 2 (Mean ± SD)	Difference (95% CI)	Test	*p*-Value
Fasting blood glucose, mg/dL (same STZ dams; *n* = 6)	GD0: 100.3 ± 5.5	GD7: 336.3 ± 51.7	+236.0 (182.6 to 289.4)	Paired t	9.2 × 10^−5^
Body weight, g: pre-mating vs. GD0 (same STZ dams; *n* = 6)	Pre-mating: 325.8 ± 47.1	GD0: 393.3 ± 54.6	+67.5 (54.6 to 80.4)	RM-ANOVA, Šidák	<0.001
Body weight, g: GD0 vs. GD7 (same STZ dams; *n* = 6)	GD0: 393.3 ± 54.6	GD7: 326.7 ± 44.0	−66.7 (−83.3 to −50.0)	RM-ANOVA, Šidák	<0.001
Body weight, g: pre-mating vs. GD7 (same STZ dams; *n* = 6)	Pre-mating: 325.8 ± 47.1	GD7: 326.7 ± 44.0	+0.8 (−10.7 to 12.3)	RM-ANOVA, Šidák	0.99
MFF, BeWo lysate, pg/mL (*n* = 3 cultures per group)	Control: 48.21 ± 0.41	Test: 71.40 ± 2.24	+23.2 (19.2 to 27.2)	Šidák/Welch	<0.001/0.0024
MFF, GD7 serum, pg/mL (*n* = 3 dams per group)	Vehicle: 45.30 ± 1.53	STZ: 63.81 ± 2.27	+18.5 (14.5 to 22.5)	Šidák/Welch	<0.001/6.2 × 10^−4^

Glucose and body weight are within-animal comparisons in the six STZ-treated dams. Glucose: paired *t*-test (GD0 vs. GD7). Body weight: repeated-measures one-way ANOVA across pre-mating, GD0 and GD7 (F(2,10) = 193.2; Greenhouse–Geisser-corrected *p* = 1.6 × 10^−7^) with Šidák-adjusted paired comparisons and 95% CIs. MFF ELISA: one-way ANOVA across the four groups with Šidák correction (primary analysis) and, because lysate and serum are different matrices, Welch’s *t*-test within each matrix (sensitivity analysis). Ninety-five percent CI of the difference: paired *t*-test for glucose; Šidák-adjusted for body weight and, from the one-way ANOVA (GraphPad Prism), for MFF. Serum MFF is relative immunoreactivity against the human-calibrated kit (Section 2.6).

## Data Availability

The mass-spectrometry raw data will be deposited to the ProteomeXchange Consortium via the PRIDE partner repository [75] upon acceptance and are available from the corresponding authors on reasonable request in the meantime. All other data generated or analysed during this study are included in this published article and its Supplementary Files, comprising the full differential-abundance tables with Benjamini–Hochberg adjusted *p*-values, the three-group re-analysis, the complete KEGG and Gene Ontology enrichment results, the condition-unique protein lists, and the in vivo and ELISA source data including sample absorbances and the calibration-curve parameters.

## Supplementary Materials

The following supporting information can be downloaded at: https://www.mdpi.com/article/10.3390/cells15212037/s1, Table S1, full differential-abundance table (Test vs. Control) with Benjamini–Hochberg-adjusted *p*-values; Table S2, full differential-abundance table (Test+VEGF vs. Control); Table S3, condition-unique proteins; Table S4, complete KEGG enrichment; Table S5, complete Gene Ontology enrichment (Test vs. Control); Table S5b, complete Gene Ontology enrichment (Test+VEGF vs. Control); Table S6, in vivo blood-glucose, body-weight and MFF ELISA source data including sample absorbances and the 4PL calibration fit; Table S7, three-group re-analysis of all nine samples with prespecified contrasts (Test vs. Control, Test+VEGF vs. Control and Test+VEGF vs. Test); Table S8, individual-dam glucose and body-weight data and ELISA effect sizes; Table S9, hypertonicity- and stress-responsive proteins.

## Abbreviations

GDM: gestational diabetes mellitus; MFF: mitochondrial fission factor; DRP1: dynamin-related protein 1; OMM: outer mitochondrial membrane; MCU: mitochondrial calcium uniporter; PDH: pyruvate dehydrogenase; PDK: pyruvate dehydrogenase kinase; EV: extracellular vesicle; LC-MS/MS: liquid chromatography–tandem mass spectrometry; LFQ: label-free quantification; DAP: differentially abundant protein; FDR: false-discovery rate; PCA: principal-component analysis; KEGG: Kyoto Encyclopedia of Genes and Genomes; GO: Gene Ontology; BP: biological process; MF: molecular function; CC: cellular component; STZ: streptozotocin; GD: gestational day; ELISA: enzyme-linked immunosorbent assay; 4PL: four-parameter logistic; VEGF: vascular endothelial growth factor; 2-NBDG: 2-(N-(7-nitrobenz-2-oxa-1,3-diazol-4-yl)amino)-2-deoxyglucose; IAEC: Institutional Animal Ethics Committee; CPCSEA: Committee for the Purpose of Control and Supervision of Experiments on Animals; FIS1: mitochondrial fission 1 protein; MFN1/MFN2: mitofusin 1/2; OPA1: optic atrophy 1 (dynamin-like GTPase); MICU1: mitochondrial calcium uptake 1; GYS1: glycogen synthase 1; BH: Benjamini–Hochberg; CI: confidence interval; FC: fold change; SD: standard deviation; AMPK: AMP-activated protein kinase; PKA: protein kinase A; GLUT1: glucose transporter 1; HAPO: Hyperglycaemia and Adverse Pregnancy Outcome; HOMA-IR: homeostasis model assessment of insulin resistance; SHBG: sex hormone-binding globulin.

## References

[1] Barbour L.A., McCurdy C.E., Hernandez T.L., Kirwan J.P., Catalano P.M., Friedman J.E. (2007). Cellular mechanisms for insulin resistance in normal pregnancy and gestational diabetes. Diabetes Care.

[2] Nguyen-Ngo C., Jayabalan N., Salomon C., Lappas M. (2019). Molecular pathways disrupted by gestational diabetes mellitus. J. Mol. Endocrinol..

[3] Wang H., Li N., Chivese T., Werfalli M., Sun H., Yuen L., Hoegfeldt C.A., Elise Powe C., Immanuel J., Karuranga S. (2022). IDF Diabetes Atlas: Estimation of global and regional gestational diabetes mellitus prevalence for 2021. Diabetes Res. Clin. Pract..

[4] McIntyre H.D., Catalano P., Zhang C., Desoye G., Mathiesen E.R., Damm P. (2019). Gestational diabetes mellitus. Nat. Rev. Dis. Primers.

[5] Catalano P.M., McIntyre H.D., Cruickshank J.K., McCance D.R., Dyer A.R., Metzger B.E., Lowe L.P., Trimble E.R., Coustan D.R., Hadden D.R. (2012). The hyperglycemia and adverse pregnancy outcome study: Associations of GDM and obesity with pregnancy outcomes. Diabetes Care.

[6] Huhn E.A., Rossi S.W., Hoesli I., Göbl C.S. (2018). Controversies in screening and diagnostic criteria for gestational diabetes in early and late pregnancy. Front. Endocrinol..

[7] Kim S.M., Park J.S., Norwitz E.R., Lee S.M., Kim B.J., Park C.-W., Jun J.K., Kim C.-W., Syn H.C. (2012). Identification of proteomic biomarkers in maternal plasma in the early second trimester that predict the subsequent development of gestational diabetes. Reprod. Sci..

[8] Ravnsborg T., Svaneklink S., Andersen L.L.T., Larsen M.R., Jensen D.M., Overgaard M. (2019). First-trimester proteomic profiling identifies novel predictors of gestational diabetes mellitus. PLoS ONE.

[9] Chu S.Y., Callaghan W.M., Kim S.Y., Schmid C.H., Lau J., England L.J., Dietz P.M. (2007). Maternal obesity and risk of gestational diabetes mellitus. Diabetes Care.

[10] Schoenaker D.A.J.M., Mishra G.D., Callaway L.K., Soedamah-Muthu S.S. (2016). The role of energy, nutrients, foods, and dietary patterns in the development of gestational diabetes mellitus: A systematic review of observational studies. Diabetes Care.

[11] Plows J.F., Stanley J.L., Baker P.N., Reynolds C.M., Vickers M.H. (2018). The pathophysiology of gestational diabetes mellitus. Int. J. Mol. Sci..

[12] Roberts K.A., Riley S.C., Reynolds R.M., Barr S., Evans M., Statham A., Hor K., Jabbour H., Norman J., Denison F. (2011). Placental structure and inflammation in pregnancies associated with obesity. Placenta.

[13] Radaelli T., Varastehpour A., Catalano P., Hauguel-de Mouzon S. (2003). Gestational diabetes induces placental genes for chronic stress and inflammatory pathways. Diabetes.

[14] Hulme C.H., Stevens A., Dunn W., Heazell A.E.P., Hollywood K., Begley P., Westwood M., Myers J.E. (2018). Identification of the functional pathways altered by placental cell exposure to high glucose. Sci. Rep..

[15] Coughlan M.T., Vervaart P.P., Permezel M., Georgiou H., Rice G. (2004). Altered placental oxidative stress status in gestational diabetes mellitus. Placenta.

[16] Sivitz W.I., Yorek M.A. (2010). Mitochondrial dysfunction in diabetes. Antioxid. Redox Signal..

[17] Jheng H.F., Tsai P.J., Guo S.M., Kuo L.-H., Chang C.-S., Su I.-J., Chang C.-R., Tsai Y.-S. (2012). Mitochondrial fission contributes to mitochondrial dysfunction and insulin resistance in skeletal muscle. Mol. Cell Biol..

[18] Zhou Y., Zhang M., Wang Y. (2021). Mitochondrial fission in metabolic diseases. Front. Physiol..

[19] Fisher J.J., Bartho L.A., Perkins A.V., Holland O.J. (2020). Placental mitochondria and reactive oxygen species in the physiology and pathophysiology of pregnancy. Clin. Exp. Pharmacol. Physiol..

[20] Otera H., Wang C., Cleland M.M., Setoguchi K., Yokota S., Youle R.J., Mihara K. (2010). Mff is an essential factor for mitochondrial recruitment of Drp1 during mitochondrial fission in mammalian cells. J. Cell Biol..

[21] Gandre-Babbe S., van der Bliek A.M. (2008). The novel tail-anchored membrane protein Mff controls mitochondrial and peroxisomal fission in mammalian cells. Mol. Biol. Cell.

[22] Liu R., Chan D.C. (2015). The mitochondrial fission receptor Mff selectively recruits oligomerized Drp1. Mol. Biol. Cell.

[23] Losón O.C., Song Z., Chen H., Chan D.C. (2013). Fis1, Mff, MiD49, and MiD51 mediate Drp1 recruitment in mitochondrial fission. Mol. Biol. Cell.

[24] Toyama E.Q., Herzig S., Courchet J., Lewis T.L., Losón O.C., Hellberg K., Young N.P., Chen H., Polleux F., Chan D.C. (2016). AMP-activated protein kinase mediates mitochondrial fission in response to energy stress. Science.

[25] Yu T., Robotham J.L., Yoon Y. (2006). Increased production of reactive oxygen species in hyperglycemic conditions requires dynamic change of mitochondrial morphology. Proc. Natl. Acad. Sci. USA.

[26] Fisher J.J., Vanderpeet C.L., Bartho L.A., McKeating D.R., Cuffe J.S.M., Holland O.J., Perkins A.V. (2021). Mitochondrial dysfunction in placental trophoblast cells experiencing gestational diabetes mellitus. J. Physiol..

[27] Abbade J., Klemetti M.M., Farrell A., Ermini L., Gillmore T., Sallais J., Tagliaferro A., Post M., Caniggia I. (2020). Increased placental mitochondrial fusion in gestational diabetes mellitus: An adaptive mechanism to optimize feto-placental metabolic homeostasis?. BMJ Open Diabetes Res. Care.

[28] Wei L., Fang C., Jiang Y., Zhang H., Gao P., Zhou X., Zhu S., Du Y., Su R., Guo L. (2025). The role of placental MFF-mediated mitochondrial fission in gestational diabetes mellitus. Diabetes Metab. Syndr. Obes..

[29] Salomon C., Scholz-Romero K., Sarker S., Sweeney E., Kobayashi M., Correa P., Longo S., Duncombe G., Mitchell M.D., Rice G.E. (2016). Gestational diabetes mellitus is associated with changes in the concentration and bioactivity of placenta-derived exosomes in maternal circulation across gestation. Diabetes.

[30] Tannetta D., Dragovic R., Alyahyaei Z., Southcombe J. (2014). Extracellular vesicles and reproduction–promotion of successful pregnancy. Cell Mol. Immunol..

[31] Mitchell M.D., Peiris H.N., Kobayashi M., Koh Y.Q., Duncombe G., Illanes S.E., Rice G.E., Salomon C. (2015). Placental exosomes in normal and complicated pregnancy. Am. J. Obstet. Gynecol..

[32] Orendi K., Kivity V., Sammar M., Grimpel Y., Gonen R., Meiri H., Lubzens E., Huppertz B. (2011). Placental and trophoblastic in vitro models to study preventive and therapeutic agents for preeclampsia. Placenta.

[33] Sharma A.K., Nayak N.R., Jena M.K. (2024). An in vitro model to study placental functions in gestational diabetes mellitus. Trends Sci..

[34] Jayabalan N., Lai A., Nair S., Guanzon D., Scholz-Romero K., Palma C., McIntyre H.D., Lappas M., Salomon C. (2019). Quantitative proteomics by SWATH-MS suggest an association between circulating exosomes and maternal metabolic changes in gestational diabetes mellitus. Proteomics.

[35] Roverso M., Brioschi M., Banfi C., Visentin S., Burlina S., Seraglia R., Traldi P., Lapolla A. (2016). A preliminary study on human placental tissue impaired by gestational diabetes: A comparison of gel-based versus gel-free proteomics approaches. Eur. J. Mass Spectrom..

[36] Liu B., Xu Y., Voss C., Qiu F.-H., Zhao M.-Z., Liu Y.-D., Nie J., Wang Z.-L. (2012). Altered protein expression in gestational diabetes mellitus placentas provides insight into insulin resistance and coagulation/fibrinolysis pathways. PLoS ONE.

[37] Lapolla A., Traldi P. (2022). Proteomic approaches in the study of placenta of pregnancy complicated by gestational diabetes mellitus. Biomedicines.

[38] Zhao C., Wang F., Wang P., Ding H., Huang X., Shi Z. (2015). Early second-trimester plasma protein profiling using multiplexed isobaric tandem mass tag (TMT) labeling predicts gestational diabetes mellitus. Acta Diabetol..

[39] Sriboonvorakul N., Hu J., Boriboonhirunsarn D., Ng L.L., Tan B.K. (2022). Proteomics studies in gestational diabetes mellitus: A systematic review and meta-analysis. J. Clin. Med..

[40] Ritchie M.E., Phipson B., Wu D., Hu Y., Law C.W., Shi W., Smyth G.K. (2015). limma powers differential expression analyses for RNA-sequencing and microarray studies. Nucleic Acids Res..

[41] Benjamini Y., Hochberg Y. (1995). Controlling the false discovery rate: A practical and powerful approach to multiple testing. J. R. Stat. Soc. B.

[42] Huang D.W., Sherman B.T., Lempicki R.A. (2009). Systematic and integrative analysis of large gene lists using DAVID bioinformatics resources. Nat. Protoc..

[43] Kanehisa M., Furumichi M., Sato Y., Ishiguro-Watanabe M., Tanabe M. (2021). KEGG: Integrating viruses and cellular organisms. Nucleic Acids Res..

[44] Ashburner M., Ball C.A., Blake J.A., Botstein D., Butler H., Cherry J.M., Davis A.P., Dolinski K., Dwight S.S., Eppig J.T. (2000). Gene Ontology: Tool for the unification of biology. Nat. Genet..

[45] Pasek R.C., Gannon M. (2013). Advancements and challenges in generating accurate animal models of gestational diabetes mellitus. Am. J. Physiol. Endocrinol. Metab..

[46] Furman B.L. (2015). Streptozotocin-induced diabetic models in mice and rats. Curr. Protoc. Pharmacol..

[47] Jawerbaum A., White V. (2010). Animal models in diabetes and pregnancy. Endocr. Rev..

[48] FDA-NIH Biomarker Working Group (2016). BEST (Biomarkers, EndpointS, and Other Tools) Resource.

[49] Fakonti G., Byford A.R., Scott E.M., Holder B., Forbes K. (2026). Decoding the molecular landscape of the placenta in maternal diabetes: A systematic review of high-throughput data. J. Mol. Endocrinol..

[50] Easton Z.J.W., Luo X., Li L., Regnault T.R.H. (2023). The impact of hyperglycemia upon BeWo trophoblast cell metabolic function: A multi-OMICS and functional metabolic analysis. PLoS ONE.

[51] Cox J., Mann M. (2008). MaxQuant enables high peptide identification rates, individualized ppb-range mass accuracies and proteome-wide protein quantification. Nat. Biotechnol..

[52] Tyanova S., Temu T., Cox J. (2016). The MaxQuant computational platform for mass spectrometry-based shotgun proteomics. Nat. Protoc..

[53] Percie du Sert N., Hurst V., Ahluwalia A., Alam S., Avey M.T., Baker M., Browne W.J., Clark A., Cuthill I.C., Dirnagl U. (2020). The ARRIVE guidelines 2.0: Updated guidelines for reporting animal research. PLoS Biol..

[54] Kamer K.J., Mootha V.K. (2015). The molecular era of the mitochondrial calcium uniporter. Nat. Rev. Mol. Cell Biol..

[55] Sugden M.C., Holness M.J. (2003). Recent advances in mechanisms regulating glucose oxidation at the level of the pyruvate dehydrogenase complex by PDKs. Am. J. Physiol. Endocrinol. Metab..

[56] Cox B., Tsamou M., Vrijens K., Neven K.Y., Winckelmans E., de Kok T.M., Plusquin M., Nawrot T.S. (2015). A co-expression analysis of the placental transcriptome in association with maternal smoking. Sci. Rep..

[57] Poidatz D., Dos Santos E., Gronier H., Vialard F., Maury B., De Mazancourt P., Dieudonné M.-N. (2015). Trophoblast syncytialisation necessitates mitochondrial function through estrogen-related receptor-γ activation. Mol. Hum. Reprod..

[58] Bucher M., Kadam L., Ahuna K., Myatt L. (2021). Differences in glycolysis and mitochondrial respiration between cytotrophoblast and syncytiotrophoblast in-vitro: Evidence for sexual dimorphism. Int. J. Mol. Sci..

[59] Chang C.R., Blackstone C. (2007). Cyclic AMP-dependent protein kinase phosphorylation of Drp1 regulates its GTPase activity and mitochondrial morphology. J. Biol. Chem..

[60] Cribbs J.T., Strack S. (2007). Reversible phosphorylation of Drp1 by cyclic AMP-dependent protein kinase and calcineurin regulates mitochondrial fission and cell death. EMBO Rep..

[61] Holland O.J., Hickey A.J.R., Alvsaker A., Moran S., Hedges C., Chamley L.W., Perkins A.V. (2017). Changes in mitochondrial respiration in the human placenta over gestation. Placenta.

[62] Muralimanoharan S., Maloyan A., Mele J., Guo C., Myatt L., Myatt L. (2012). MIR-210 modulates mitochondrial respiration in placenta with preeclampsia. Placenta.

[63] Wang W., Li J., Mu L., Bai Y., Zhu M., Zhao Y., Hu S., Wang J., Shao P., Su X. (2025). Vitamin K2 alleviates metabolic dysfunction-associated steatotic liver disease through mitochondrial dysfunction modulation via gut microbiota. Int. J. Vitam. Nutr. Res..

[64] Chan D.C. (2020). Mitochondrial dynamics and its involvement in disease. Annu. Rev. Pathol..

[65] Lee C.A., Chin L.S., Li L. (2018). Hypertonia-linked protein Trak1 functions with mitofusins to promote mitochondrial tethering and fusion. Protein Cell.

[66] Franco A., Walton C.E., Dang X. (2022). Mitochondria clumping vs. mitochondria fusion in CMT2A diseases. Life.

[67] Lowe W.L., Scholtens D.M., Lowe L.P., Kuang A., Nodzenski M., Talbot O., Catalano P.M., Linder B., Brickman W.J., Clayton P. (2018). Association of gestational diabetes with maternal disorders of glucose metabolism and childhood adiposity. JAMA.

[68] Godfrey K.M., Reynolds R.M., Prescott S.L., Nyirenda M., Jaddoe V.W.V., Eriksson J.G., Broekman B.F.P. (2017). Influence of maternal obesity on the long-term health of offspring. Lancet Diabetes Endocrinol..

[69] Jansson T., Powell T.L. (2007). Role of the placenta in fetal programming: Underlying mechanisms and potential interventional approaches. Clin. Sci..

[70] Castillo-Castrejon M., Powell T.L. (2017). Placental nutrient transport in gestational diabetic pregnancies. Front. Endocrinol..

[71] Castillo-Castrejon M., Jansson T., Powell T.L. (2019). No evidence of attenuation of placental insulin-stimulated Akt phosphorylation and amino acid transport in maternal obesity and gestational diabetes mellitus. Am. J. Physiol. Endocrinol. Metab..

[72] Lappas M. (2014). Activation of inflammasomes in adipose tissue of women with gestational diabetes. Mol. Cell Endocrinol..

[73] Troncoso F., Acurio J., Herlitz K., Aguayo C., Bertoglia P., Guzman-Gutierrez E., Loyola M., Gonzalez M., Rezgaoui M., Desoye G. (2017). Gestational diabetes mellitus is associated with increased pro-migratory activation of VEGFR2 and reduced expression of VEGFR1. PLoS ONE.

[74] Harris L.K., Crocker I.P., Baker P.N., Aplin J.D., Westwood M. (2011). IGF2 actions on trophoblast in human placenta are regulated by the insulin-like growth factor 2 receptor. Biol. Reprod..

[75] Perez-Riverol Y., Bai J., Bandla C., García-Seisdedos D., Hewapathirana S., Kamatchinathan S., Kundu D.J., Prakash A., Frericks-Zipper A., Eisenacher M. (2022). The PRIDE database resources in 2022: A hub for mass spectrometry-based proteomics evidences. Nucleic Acids Res..

